# Enhanced Oxide Ion Diffusion by Lanthanum Substitution in the Palmierite Sr_3–3*x*_La_2*x*_V_2_O_8_ via Increased Tetrahedral Distortion and Cation Vacancies

**DOI:** 10.1021/acs.chemmater.5c01856

**Published:** 2025-09-19

**Authors:** Victoria Watson, Ying Zhou, Ronald I. Smith, Sacha Fop, Yi Sun, Zongping Shao, San Ping Jiang, Oscar J. B. Ballantyne, James A. Dawson, Abbie C. Mclaughlin

**Affiliations:** † Advanced Centre for Energy and Sustainability (ACES), The Chemistry Department, 1019 University of Aberdeen, Aberdeen AB24 3UE, U.K.; ‡ Chemistry − School of Natural and Environmental Sciences, 5994 Newcastle University, Newcastle NE1 7RU, U.K.; § ISIS Neutron and Muon Source, Rutherford Appleton Laboratory, Chilton, Didcot OX11 0QX, U.K.; ∥ WA School of Mines: Minerals, Energy and Chemical Engineering, 1649 Curtin University, Perth WA 6102, Australia; ⊥ Foshan Xianhu Laboratory of the Advanced Energy Science and Technology Guangdong Laboratory, Foshan 528216, China

## Abstract

Promising ionic conductivity has previously been reported in the palmierite oxide Sr_3_V_2_O_8_. Oxide-ion diffusion within this system occurs via the “cog-wheel” mechanism where rotation of VO_4_ and oxygen deficient VO_3_ units result in the formation of V_2_O_7_ dimers which continuously break and reform. During this process an oxide ion from a VO_4_ tetrahedra can move to the vacant site of the VO_3_ group, facilitating the movement of oxide-ions throughout the structure. Here, we report the electrical and structural properties of the series Sr_3–3*x*_La_2*x*_V_2_O_8_ (*x* = 0.00–0.25). Combined neutron diffraction and atomistic simulations reveal that substituting Sr^2+^ with La^3+^ results in ordered cation vacancies, an elongation of the apical V–O1 bond and an increase in the distortion of the VO_4_ tetrahedra, which together enhances the cog-wheel-like rotational dynamics of the VO_4_ tetrahedra that mediate oxide ion transport. Molecular dynamics simulations further indicate that La-substitution facilitates the formation of a continuous diffusion network, leading to improved oxide ion conductivity so that Sr_2.55_La_0.3_V_2_O_8_ (*x* = 0.15) exhibits the highest bulk conductivity of 7.64 × 10^–4^ S cm ^–1^ at 700 °C, an order of magnitude higher than Sr_3_V_2_O_8_. The results demonstrate that palmierites are highly flexible to doping strategies for improving the oxide ion conductivity.

## Graphical abstract

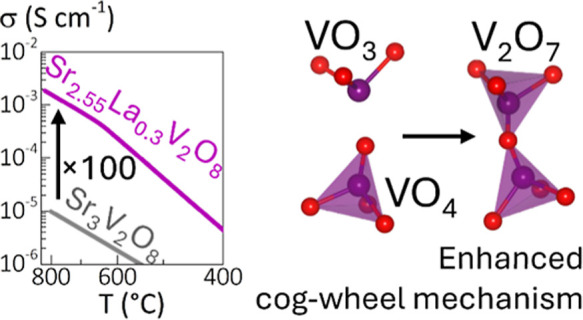

## Introduction

Oxide-ion conductors are important materials for applications in hydrogen-based technologies such as solid oxide fuel cells (SOFCs) and electrolyzers (SOECs). These technologies exhibit high efficiencies and provide a promising way of utilizing hydrogen for green energy production as well as generating green hydrogen via water electrolysis. At present, the greatest challenge associated with SOFCs is the high operating temperatures required to produce sufficient ionic conduction through commercially available electrolytes. The majority of SOFCs use yttria stabilized zirconia (YSZ) as the electrolyte, which only exhibits significant conductivity at temperatures >700 °C.^1–5^ This poses technical challenges, such as poor durability of components and longer start-up times. Therefore, developing new materials which exhibit high conductivities at lower temperatures (300–600 °C) is crucial.^1–5^

The oxide-ion conductivity of a material is dependent on its crystal structure with characteristics such as structural disorder, oxygen vacancies and oxygen interstitials playing an important role in achieving high conductivities. So far ionic conduction has been reported in several different crystal systems including fluorite-type oxides (AO_2_), La_2_Mo_2_O_9_ (LAMOX) materials, apatite-type oxides, melilite-structured gallates and perovskites.^1,6,7^ The perovskite family appears to be among the most promising for new oxide-ion conductors due to its ability to adopt different structural derivatives. Sizeable ionic conduction has previously been reported for cubic perovskite materials (ABO_3_) including La_0.9_Sr_0.1_Ga_0.8_Mg_0.2_O_3‑δ_ (LGSM) and Na_0.5_Bi_0.5_TiO_3_ (NBT).^1,8–10^ More recently significant ionic conductivity has been reported in several hexagonal perovskite derivatives such as Ba_3_NbMoO_8.5_ and Ba_7_Nb_4_MoO_20_ that are composed of perovskite and palmierite-like (P-L) layers.^11–13^ Ba_3_NbMoO_8.5_ exhibits a bulk oxide-ion conductivity of 2.2 × 10^–3^ S cm^–1^ at 600 °C and has an average structure that can be described as a disordered hybrid of the 9R hexagonal perovskite (A_3_B_3_O_9_) and palmierite (A_3_B_2_O_8_).^12^ The palmierite structure is formed from the 9R polytype by replacing the AO_3_ cubic layer with an oxygen deficient AO_2_ layer that results in isolated tetrahedral units separated by octahedral vacancies. Ba_7_Nb_4_MoO_20_ can be described as a cation-deficient 7H hexagonal perovskite derivative formed by the intergrowth of 12R perovskite layers and P-L layers (Figure 1). It exhibits a bulk conductivity of ∼1.9 × 10^–3^ S cm^–1^ at 510 °C under dry air and ∼4.0 × 10^–3^ S cm^–1^ at 510 °C under wet air so that it supports both oxide-ion and proton conduction.^13^ In both materials ionic conduction predominately occurs within the palmierite-like layers.

**Figure 1:**
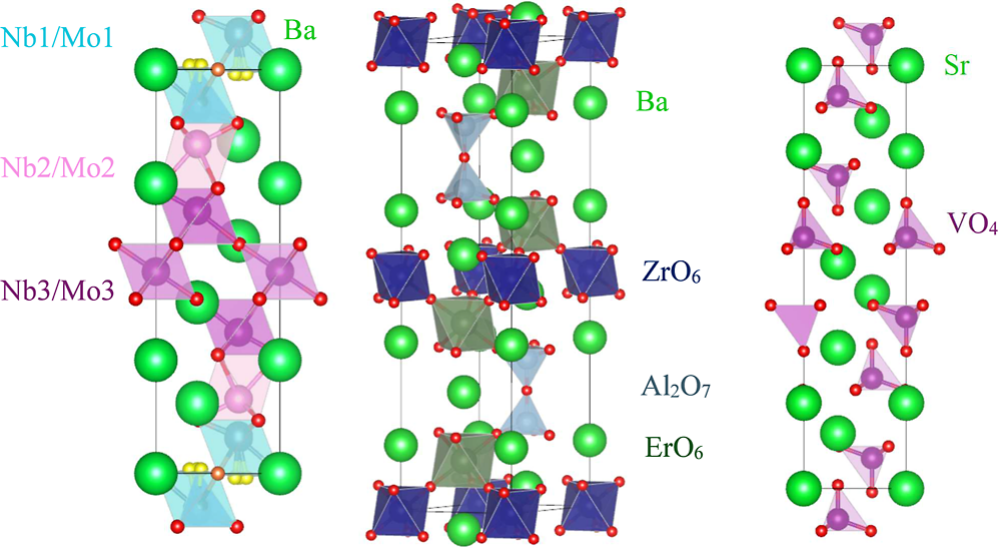
Crystal structures of hexagonal perovskite derivative oxides. From left to right Ba_7_Nb_4_MoO_20_ (7H), Ba_5_Er_2_Al_2_ZrO_13_ (10H) and Sr_3_V_2_O_8_ (palmierite).

Other hexagonal perovskites that support high proton conductivity include Ba_5_Er_2_Al_2_ZrO_13_ (Figure 1) which can be described as a 10H hexagonal perovskite derivative that is formed from the stacking of cubic (BaO_3_) layers and oxygen deficient hexagonal (BaO) layers.^14^ These oxygen deficient BaO layers result in the formation of tetrahedral units which form Al_2_O_7_ units when connected by a shared apical oxygen site. Under dry air the conductivity of Ba_5_Er_2_Al_2_ZrO_13_ is electronic p-type, however under humidified air it exhibits a significant proton conductivity of ∼3 × 10^–3^ S cm^–1^ at 500 °C.^14,15^ The intrinsic oxygen vacancies of the BaO layer are vital to the proton conduction mechanism of Ba_5_Er_2_Al_2_ZrO_13_ as water is absorbed onto these vacancies while the proton sites are located on the apical oxygen site of the Al_2_O_7_ unit.^14,15^

The crystal structure of the palmierite oxides A_3_V_2_O_8_ (A = Sr, Ba) is shown in Figure 1. The ionic conductivity of A_3_V_2_O_8_ (A = Sr, Ba) has also been investigated to assess if a structure composed of only palmierite layers can exhibit both oxide ion and proton conductivity.^16^ Sr_3_V_2_O_8_ was found to exhibit a higher ionic conductivity than Ba_3_V_2_O_8_, displaying a bulk oxide-ion conductivity of 7.41 × 10^–5^ S cm^–1^ under dry air and a bulk proton conductivity of 1.6 × 10^–4^ S cm^–1^ at 700 °C.^16^ While this shows that palmierites exhibit both sizable oxide-ion and proton conductivity, the magnitude of conductivity is relatively low compared to other hexagonal perovskite derivatives. The migration of oxide ions in Sr_3_V_2_O_8_ occurs through a “cog-wheel” mechanism in which the cooperative rotation of VO_4_ and oxygen-deficient VO_3_ units result in the formation of transient V_2_O_7_ dimers that repeatedly form and dissociate. Oxygen vacancies on the VO_4_ tetrahedra arise from a slight oxygen non stoichiometry generated during the high temperature synthesis. The vacancies are not visible by neutron diffraction but are confirmed by AIMD simulations.^16^ However, it has recently been reported that introducing oxygen interstitials into the palmierite structure results in a significant increase in the oxide-ion conductivity.^17^ Ba_3_Ti_0.9_Mo_1.1_O_8.1_ is reported to exhibit a high total oxide ion conductivity of 3.96 × 10^–3^ S cm^–1^ at 600 °C – 2 orders of magnitude higher than that reported previously for palmierites.^17^ By replacing V^5+^ with Ti^4+^ and Mo^6+^ and altering the Ti/Mo ratio, interstitial oxygen was introduced into the structure resulting in a change in the oxide-ion migration mechanism from the cog-wheel mechanism previously reported for A_3_V_2_O_8_ (A = Ba, Sr) to an interstitialcy mechanism. This change causes the significant increase in the oxide-ion conductivity.

It is not yet known if it is possible to enhance the oxide ion conductivity via the cog-wheel mechanism in palmierites. In this study, we investigate the effect of lanthanum (La^3+^) substitution on the strontium (Sr^2+^) site on the structural and electrical properties of Sr_3_V_2_O_8._ Six different compositions Sr_3–3*x*_La_2*x*_V_2_O_8_ (*x* = 0.00, 0.05, 0.10, 0.15, 0.20, 0.25) were synthesized and characterized by X-ray and powder neutron diffraction, AC impedance spectroscopy, and density functional theory and molecular dynamics calculations. The results show a significant increase in the oxide-ion conductivity upon lanthanum doping, with Sr_2.55_La_0.3_V_2_O_8_ (*x* = 0.15) exhibiting the highest bulk conductivity of 7.64 × 10^–4^ S cm^–1^ at 700 °C. This demonstrates that substitution of La^3+^ onto the Sr^2+^ site of Sr_3_V_2_O_8_ significantly increases the oxide-ion conductivity by an order of magnitude so that the palmierite is a highly promising platform for the development of efficient oxide-ion conductors.

## Results

The X-ray diffraction patterns for the Sr_3–3*x*_La_2*x*_V_2_O_8_ series are shown in Figure S1. All of the patterns could be indexed with the space group *R*3̅*m* in agreement with the structural model previously reported for Sr_3_V_2_O_8_.^16^ Samples were found to be phase pure up to *x* = 0.25 where a small amount of La_2_O_3_ impurity began to form indicating the solid solution limit.

### Ionic Conductivity

Typical complex impedance Z* plots recorded under dry air for the Sr_2.85_La_0.10_V_2_O_8_ and Sr_2.55_La_0.30_V_2_O_8_ samples are shown in Figure 2a and Figure 2b, respectively. Both plots show three clear responses which were attributed to the bulk (∼2.9–10 pF cm^–1^), grain boundary (∼3.8–31 nF cm^–1^) and Warburg electrode response (∼1.3–36 μF) which is distinctive of ionic conductivity and indicates the presence of oxide-ion conduction.^18^

**Figure 2:**
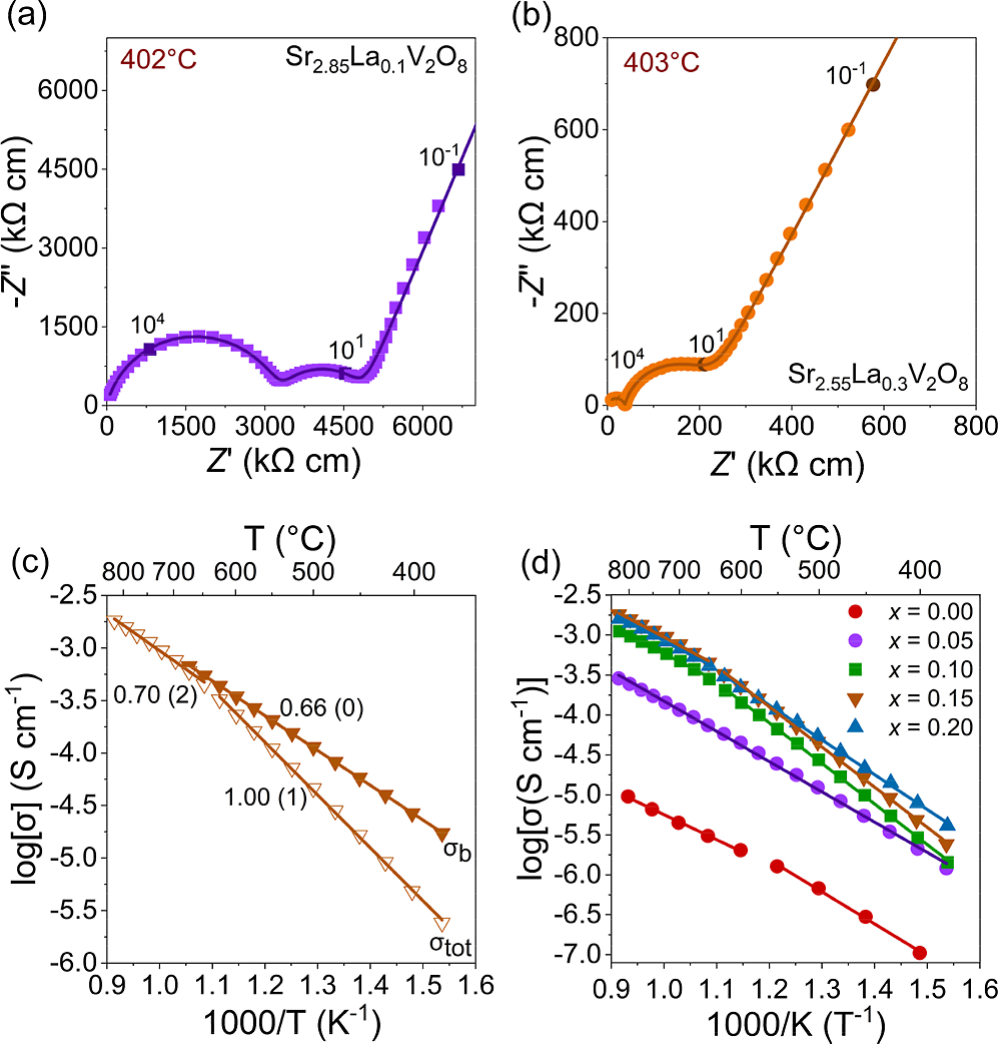
Typical complex impedance plots for (a) Sr_2.85_La_0.1_V_2_O_8_ (*x* = 0.05) collected under dry air and (b) Sr_2.55_La_0.3_V_2_O_8_ (*x* = 0.15) collected under dry air. The line represents the equivalent circuit fitting to the data. (c) Arrhenius plots of the bulk and total conductivity of Sr_2.55_La_0.3_V_2_O_8_ under dry air with the respective activation energies (in eV). (d) Arrhenius plots of the total conductivity under dry air for the Sr_3–3*x*_La_2*x*_V_2_O_8_ series. * Data obtained from ref 16.

Equivalent circuit fitting was performed to extract the individual bulk, grain boundary and electrode responses from the impedance data collected for the Sr_3–3*x*_La_2*x*_V_2_O_8_ series. Figure S2 shows the equivalent circuit employed to model the data for the Sr_3–3*x*_La_2*x*_V_2_O_8_ samples for all compositions (*x* = 0.0–0.20). At higher temperatures (>675 °C) where the grain boundary response is no longer visible, the total conductivity was extracted from the high frequency intercept of the electrode arc on the real impedance axis.

The complex impedance plot (Figure S3a) shows no significant change in the impedance under humidified air for the Sr_2.55_La_0.3_V_2_O_8_ sample indicating that the proton conduction is completely suppressed upon lanthanum doping. This can also be seen from the Arrhenius plot (Figure S3b) which shows no change in the conductivity from dry to wet air for the lanthanum doped *x* = 0.05 and *x* = 0.15 samples.

The total conductivity of Sr_2.55_ La_0.3_V_2_O_8_ as shown in Figure 2d is 7.64 × 10^–4^ S cm^–1^ at 700 °C under dry air and is more than 2 orders of magnitude higher than Sr_3_V_2_O_8_ which was previously reported to exhibit a total conductivity of 3.98 × 10^–6^ S cm^–1^ at 700 °C under dry air.

Further impedance spectroscopy measurements recorded under dry O_2_ and N_2_ reveal that the total conductivity (Figures S3c,d) is unchanged across the entire temperature range which suggests a negligible electronic component. The low-frequency Warburg signal, which is present under all gases across the entire temperature range, is consistent with oxide-ion conduction in a material with partially blocking electrodes. X-ray diffraction data recorded under different atmospheres (Figures S4–S7) show that the lanthanum doped phases are stable under dry and humidified air, O_2_ and N_2_. However, extra peaks are observed in the X-ray diffraction pattern recorded after Sr_2.55_La_0.3_V_2_O_8_ was annealed for 10 h under flowing 5% H_2_/N_2_ at temperatures ≥500 °C (Figure S8). These peaks were attributed to the formation of a La_0.5_Sr_0.5_VO_2.95_ impurity phase, indicating that the lanthanum doped phases are unstable under 5% H_2_/N_2_. Subsequently the ionic conductivity of the Sr_2.55_La_0.3_V_2_O_8_ sample under 5% H_2_/N_2_ was not measured.

### Investigation of the Crystal Structure

Initial Rietveld refinements using the X-ray diffraction data collected for the Sr_3–3*x*_La_2*x*_V_2_O_8_ series (Figure S9) were performed to determine the fractional occupancies and isotropic displacement parameters for vanadium since its scattering cross-section for neutrons is small. The Sr atoms are situated at two different Wyckoff sites; Sr1 at 3*a* and Sr2 at 6*c*. To begin with it was assumed that the La^3+^ cations occupy both Sr sites. However, initial Rietveld refinements showed the refined occupancy of the La2 site to be less than zero so that the La^3+^ cations are situated on the Sr1 site only. This is likely due to the fact that La^3+^ cations prefer the 6-fold coordination (Sr1 site) over the 10-fold coordination (Sr2 site).^19^ A constraint was placed on the Sr1/Sr2 fractional occupancies so that as they varied, the overall stoichiometry would remain the same. Substitution of La^3+^ into the structure also introduces cationic vacancies onto the Sr2 site. This is indicated in Table S1 as the Sr2 site occupancy decreases with increased La concentration. Since there is no lanthanum situated on the Sr2 site, the observed decrease in occupancy must be a result of the introduction of cationic vacancies onto the site. The V and O1 positions are situated at Wycoff sites 6*c* and the O2 position at 18 *h*. The V fractional occupancy refined to within ±1% of the full occupancy in the XRD refinement and subsequently was fixed at 1. Figure S9 shows an excellent Rietveld fit to the *R*3̅*m* model for the Sr_3_V_2_O_8_ and Sr_2.55_ La_0.3_V_2_O_8_ samples from the X-ray diffraction data. The refined atomic parameters and agreement factors obtained from the Rietveld fit to the *R*3̅*m* model from the X-ray diffraction data for the complete Sr_3–3*x*_La_2*x*_V_2_O_8_ (*x* = 0.00–0.20) series can be found in Table S1.

The structure obtained from the Rietveld refinements with room temperature X-ray diffraction data was employed as the starting model for the variable temperature neutron refinements (Tables S2–S4). The atomic displacement parameters were modeled anisotropically for all atoms except V which were set to the refined *U*_iso_ values obtained from the initial X-ray refinements. Rietveld refinement from neutron diffraction data (Tables S2 and S3) showed the refined occupancy of the La2 site to be less than zero for all compositions both at 25 and 700 °C. Therefore, the La^3+^ cations are situated on the Sr1 site only, consistent with the results from the initial Rietveld refinements using X-ray diffraction data. Tables S2 and S3 show the Sr2 site occupancy decreases with increased La concentration at both 25 and 700 °C. This is also in agreement with initial X-ray diffraction refinements where the observed decrease in the Sr2 site occupancy is attributed to the introduction of cationic vacancies onto the site. As a result, a constraint was placed on the Sr1/Sr2 fractional occupancies so that as they varied, the overall stoichiometry would remain the same. A constraint was also applied on the *U*_aniso_ values for the Sr1 and La1 positions to be the same. Both the O1 and O2 occupancies refined to within ±1% of the full occupancy suggesting that no extra oxygen is introduced into the structure upon lanthanum doping. Difference Fourier maps generated from the POLARIS neutron diffraction data also showed no evidence of residuals in scattering density that would indicate the presence of additional interstitial oxygen. Therefore, the O1 and O2 occupancies were fixed at 1. An excellent Rietveld fit was obtained to the *R*3̅*m* model at 25 and 700 °C for all *x* (Figures 3 and S10).

**Figure 3:**
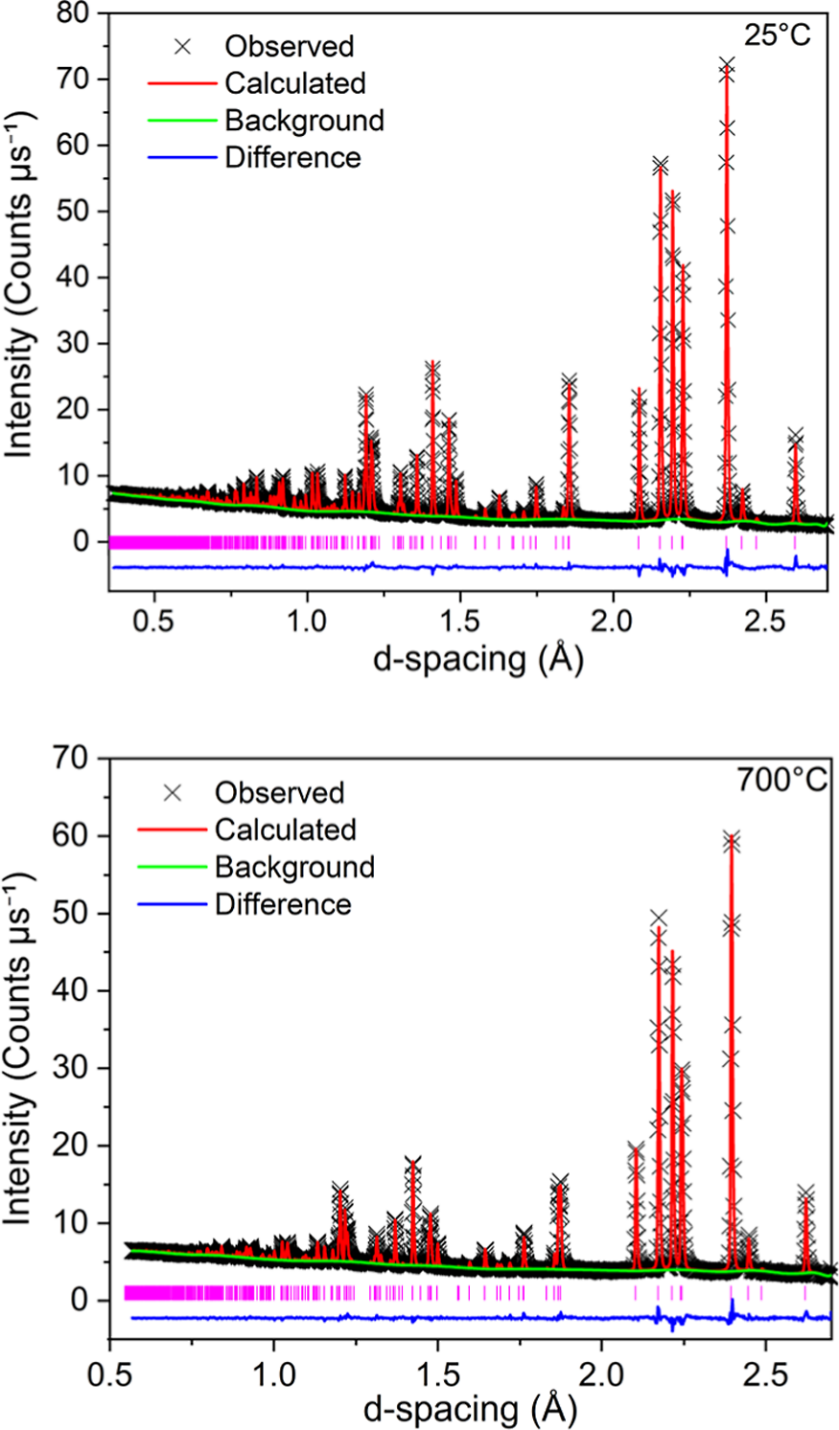
Rietveld refinement fits to the *R*3̅*m* model for Sr_2_._55_La_0.3_V_2_O_8_ at 25 °C (top) and 700 °C (bottom). Black crosses show the observed data, the red line is the Rietveld fit and the green line is the background function, the blue line shows the difference between the calculated and observed data. Vertical pink lines show the reflection positions for Sr_2.55_La_0.3_V_2_O_8._.

The statistical parameters obtained from the refinements, refined atomic positions and unit cell parameters are reported in Tables S2–S4. There is a decrease in the *c* cell parameter with increasing La doping at 25 °C as shown in Table S2. This is due to the substitution of La^3+^ cations which have smaller ionic radii (1.032 Å) than Sr^2+^ cations (1.18 Å). The unit cell parameter *a* at 25 °C increases with increasing La concentration despite its smaller ionic radius. This is likely due to the presence of cation vacancies which arise due to the substitution of La^3+^ for Sr^2+^.

The variation of selected bond lengths and angles with increasing lanthanum doping amount are reported in Tables S5–S7. The Sr–O2 bond length decreases from 2.6104(4) Å to 2.5825(5) Å at 25 °C and from 2.6329(5) Å to 2.6017(7) Å at 700 °C as *x* increases from 0.0–0.2. The apical V–O1 bond length increases with increasing La concentration from 1.634(6) Å to 1.689(8) Å at 25 °C and from 1.607(7) Å to 1.708(10) Å at 700 °C as shown in Figure 4. The V–O2 bond length simultaneously decreases from 1.729(2) Å to 1.702(3) Å at 25 °C and from 1.734(3) Å to 1.697(3) Å at 700 °C as shown in Figure 4. While both V–O bonds change upon lanthanum doping the increase in the apical V–O1 bond is more significant than the observed decrease in the V–O2 bond since the relative increase in the V–O1 bond length at 700 °C is 6.29% compared to the relative decrease in the V–O2 bond length which is only 2.13%.

**Figure 4:**
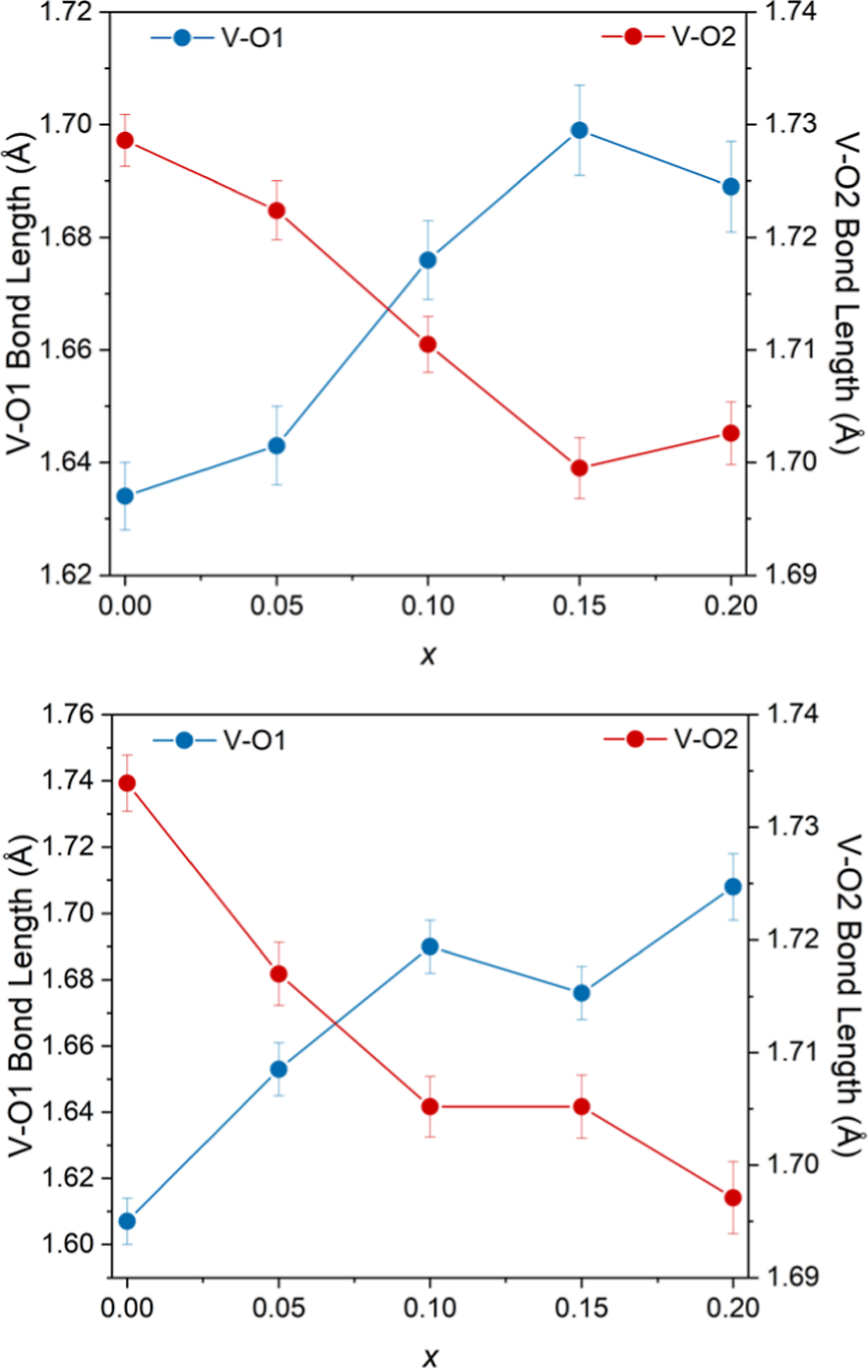
Variation of the V–O1 and V–O2 bond lengths with increasing lanthanum content at 25 °C (top) and 700 °C (bottom).

Upon increasing *x* in Sr_3–3*x*_La_2*x*_V_2_O_8_, the O1–V–O2 angle decreases from 111.1(2)° to 108.9(3)° at 25 °C while the O2–V–O2 angle simultaneously increases from 107.8(2)° to 110.0(3)°. This shows that both bond angles are becoming more ideal as they tend toward 109.5°. At 700 °C, the O1–V–O2 angle decreases from 111.4(2)° to 107.7(3)° while the O2–V–O2 angle simultaneously increases from 107.5(2)° to 111.1(3)°. The decrease in the O1–V–O2 bond angle accompanied by the increase O2–V–O2 angle indicates that the VO_4_ polyhedra are becoming more oblate.

The thermal variation of the unit cell parameters, bond lengths and angles for the Sr_2.55_La_0.3_V_2_O_8_ sample are shown in Tables S4 and S7. Upon increasing temperature, both the *a* and *c* cell parameters increase because of thermal expansion. Similarly, the Sr2–O1 and Sr1–O2 bond lengths both increase as the unit cell expands. Increasing temperature also appears to have the same effect on the O1–V–O2 and O2–V–O2 bond angles as increasing lanthanum doping, as the O1–V–O2 angle decreases from 110.44(2)° to 108.8(3)° while the O2–V–O2 angle simultaneously increases from 108.49(2)° to 110.2(3)°.

The PIEFACE software package was used to determine the minimum bounding ellipsoid for each composition in the Sr_3–3*x*_La_2*x*_V_2_O_8_ (*x* = 0–0.2) series at 25 and 700 °C. By fitting the smallest bounding ellipsoid around the VO_4_ polyhedra the polyhedral distortion σ­(*R*), ellipsoidal shape parameter (*S*) and out-of-center cation displacement (*d*) can be determined. The results are reported in Tables S8 and S9. The cation displacement (*d*) decreases with increasing lanthanum concentration at both 25 and 700 °C. This is linked to changes in the vanadium *z* position as shown in Figure S11. At 25 °C the vanadium z position decreases from 0.5962(3) for *x* = 0.00 to 0.5949 (4) for *x* = 0.20. At 700 °C the vanadium z position also decreases from 0.5971 (3) for *x* = 0.00 to 0.5934 (5) for *x* = 0.20. The polyhedral distortion σ­(R) increases from 0.0081 Å (*x* = 0.00) to 0.0129 Å (*x* = 0.20) at 25 °C, and from 0.0152 Å (*x* = 0.00) to 0.0241 Å (*x* = 0.20) at 700 °C. Hence as more lanthanum is added, the VO_4_ polyhedra become more distorted. These changes to the VO_4_ tetrahedra are observed alongside changes in the oxygen *z* positions. At 25 °C the O1 *z* position increases from 0.67755(3) at x = 0.00 to 0.67922(4) at *x* = 0.20, which causes the apical V–O1 bond to become longer with increased La concentration (Figure 4). The O2 *z* position also increases from 0.23187(2) at *x* = 0.00 to 0.23394(2) at *x* = 0.20, which combined with the decrease in the V *z* position causes the V–O2 bond to become shorter. The same trend is observed at 700 °C. Hence the polyhedral distortion is likely influenced by changes in the oxygen *z* positions caused by the introduction of both lanthanum and cationic vacancies into the structure.

For the Sr_2.55_La_0.3_V_2_O_8_ sample, as temperature is increased, the polyhedral distortion σ­(*R*) also increases from 0.0117 at 25 °C to 0.0204 Å at 700 °C (Table S10). A similar trend has previously been reported for Sr_3_V_2_O_8_ where σ­(*R*) increases with rising temperature from 0.00696 Å at 25 °C to 0.01569 at 700 °C.^20^ The thermal variation of the distortion of the VO_4_ tetrahedra has been reported to be related to the change in O1 and O2 positions.^20^ This can also be observed for the Sr_2.55_La_0.3_V_2_O_8_ sample as the O1 *z* position decreases leading to the shortening of the apical V–O1 from 1.699(8) Å at 30 °C to 1.676(8) at 700 °C. Meanwhile the O2 *x* position decreases, while both the *y* and *z* positions increase. The changes to both the O1 and O2 positions results in the VO_4_ tetrahedra becoming more distorted as temperature is increased.

The shape parameter, *S*, describes whether the minimum bounding is a perfect sphere *S* = 0, axially compressed *S* < 0 or axially stretched *S* > 0. With increasing lanthanum concentration, the shape parameter decreases from −0.01002 (*x* = 0.00) to −0.01601 (*x* = 0.20) at 25 °C, and from −0.01888 (*x* = 0.00) to −0.02970 (*x* = 0.20) at 700 °C. This means that the VO_4_ polyhedra are becoming axially compressed and therefore more oblate with increased lanthanum doping. This is in agreement with the observed decrease in the O1–V–O2 bond angle and increase in the O2–V–O2 bond angle as stated previously.

Three possible pathways for oxide-ion diffusion are reported for Sr_3_V_2_O_8_: between the O1–O1 (1), O1–O2 (2), and O2–O2 (3) positions as shown in Figure 5.^16,20^

**Figure 5:**
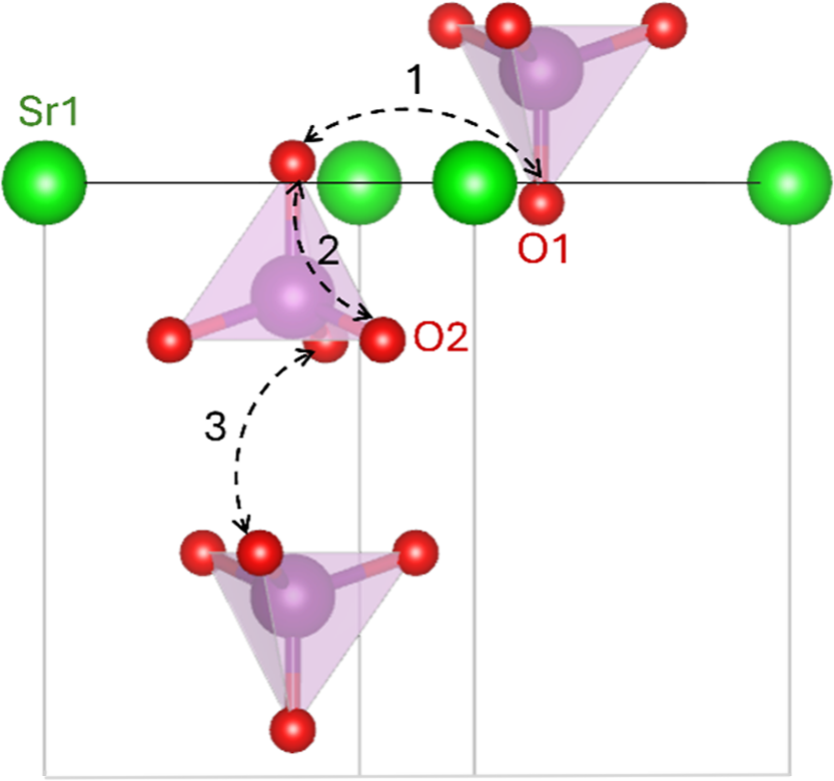
Potential migration pathways in Sr_3_V_2_O_8_ between O1–O1 (1), O1–O2 (2) and O2–O2 (3) positions.

In Sr_3_V_2_O_8_, oxide-ion migration occurs via the “cog-wheel” mechanism where rotation of VO_4_ and oxygen deficient VO_3_ units result in the formation of V_2_O_7_ dimers which continuously break and reform. During this process an oxygen atom from a VO_4_ tetrahedra can move to the vacant site of the VO_3_ group hence facilitating the movement of the oxide ions. The increase in the apical V–O1 bond length means that the bond will become weaker upon lanthanum doping, and the oxygen situated at the O1 position will be more mobile, making it easier for the oxide-ions to migrate to a vacant site. Hence the associated energy barriers decrease, and the oxide-ion conductivity increases as shown in Figure 6. Moreover, it has previously been reported that the rotation of the VO_4_ groups in A_3_V_2_O_8_ is pivotal for achieving oxide ion transport.^16^ Upon La doping in Sr_3–3*x*_La_2*x*_V_2_O_8_, the VO_4_ tetrahedra become more distorted as both *x* and temperature increase. When the VO_4_ polyhedra become distorted, the local symmetry will reduce, further lowering the energy barrier for polyhedral rotation or tilting and enhancing oxide ion migration. Furthermore, each VO_4_ tetrahedron connects to three Sr1 and seven Sr2 sites via M–O–Sr bonds. The presence of vacancies at the Sr2 site (Table S3) will further increase the flexibility of the VO_4_ tetrahedra and enhance oxide ion migration.

**Figure 6:**
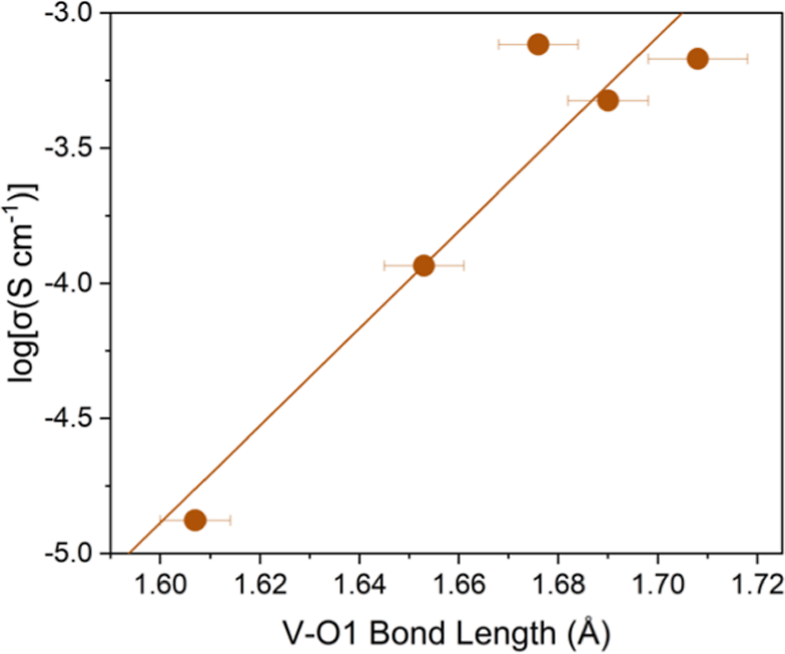
Variation of log­(σ) with increasing apical V–O1 bond length showing an almost linear increase in log­(σ) as the V–O1 bond length expands.

### Atomistic Simulations

Figure 7 presents the oxide-ion conductivity derived from MD simulations for the Sr_3–3*x*_La_2*x*_V_2_O_8_ (*x* = 0–0.2) series under dry air conditions. With La doping, the conductivity improves markedly, indicating enhanced oxide-ion mobility. This is clear from the reduction in activation energy from 0.42 eV for undoped Sr_3_V_2_O_8_ to 0.25 eV for *x* = 0.2. These results suggest that La doping effectively lowers the energy barrier for oxide-ion migration, thereby facilitating improved ionic transport. The MD simulations show that the highest conductivity is observed for *x* = 0.05 whereas experimentally this is achieved for *x* = 0.15. This discrepancy likely arises from the idealized conditions used in the simulations, which do not fully capture the complexities of real materials, such as microstructural effects or defects. Figure S12 presents the variation in V–O1 and V–O2 bond lengths as a function of La concentration in the system, as predicted by MD simulations at 700 and 1000 K. In agreement with the neutron diffraction analysis, the simulations similarly show that increasing La content leads to an increase in the V–O1 bond length and a concurrent decrease in the V–O2 bond length, with the magnitude of change slightly enhanced at higher temperatures. This consistency confirms that La doping induces a redistribution in the V–O coordination environment, a structural feature that persists across temperature and is reliably captured by both experimental and computational approaches.

**Figure 7:**
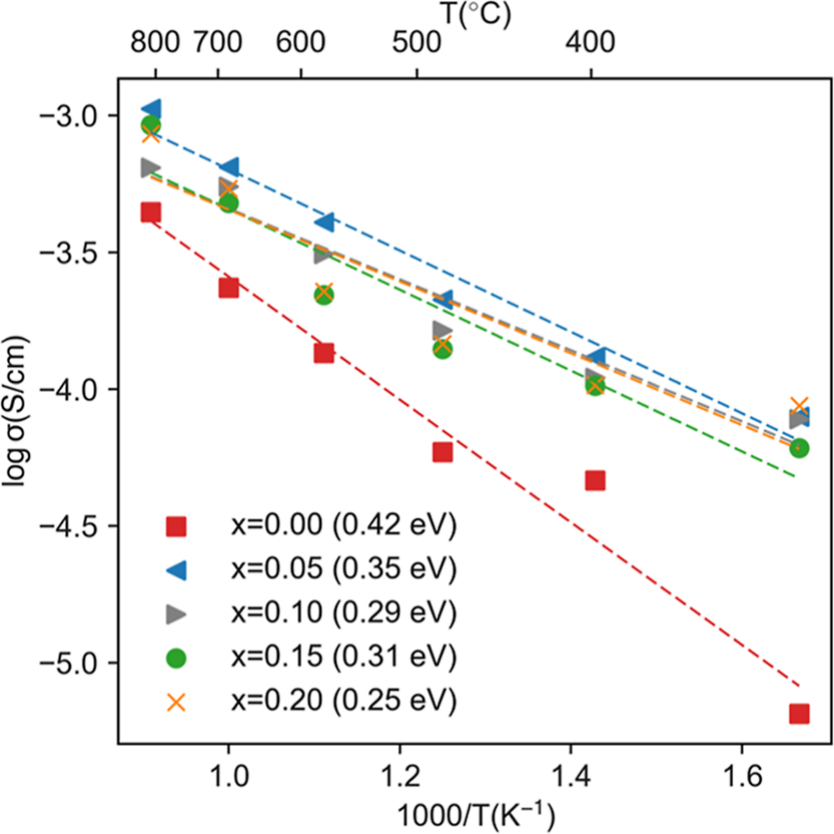
Arrhenius plot of oxide-ion conductivity for the Sr_3–3*x*_La_2*x*_V_2_O_8_ series predicted by MD simulations.

Figure 8 presents a visual analysis of the oxide-ion dynamics in Sr_3–3*x*_La_2*x*_V_2_O_8_ from the MD simulations at 700 and 1000 K. Over 10 ns, the oxide-ion trajectories show a clear increase in mobility with La doping. At 700 K, oxide-ion diffusion in the undoped system (*x* = 0) is limited, with oxide ion transitions mostly between O2–O2 and O1–O2. Upon La-substitution, additional O1–O1 transitions appear and the frequency of local transitions increases, reflecting an overall enhancement of oxide-ion dynamics rather than a strictly monotonic trend with composition. At 1000 K, thermal activation enhances all pathways, resulting in more interconnected ion migration across the structure. This is further evidenced in the calculated energy barriers. Table S13 shows that for Sr_3_V_2_O_8_, the lowest energy barriers are for the O1–O2 and O2–O2 pathways and oxide ion migration is observed mainly along *c*. With increased La doping, the O1–O1 and O2–O2 energy barriers significantly reduce (Table S11), and the trajectories become more extended and interconnected. These changes lead to more continuous and collective motion, resulting in extended, interconnected trajectories and enhanced long-range oxide-ion diffusion. Figure S13 shows the radial distribution function (*g*(*r*)) of the V–O bond in Sr_3–3*x*_La_2*x*_V_2_O_8_ for various La doping levels. The RDF data indicate that La doping affects the local V–O structure, likely due to Sr vacancy and lattice strain effects from substituting La for Sr. Even though the V–O bond distance remains centered near 1.72 Å, the peak shows slight broadening and a small change in intensity with La-substitution. These subtle features may indicate a modest increase in static or dynamic disorder in the local structure, although the effect is relatively minor. During our molecular dynamics simulations, we were able to detect transient corner-sharing V_2_O_7_ dimers (two VO_4_ tetrahedra sharing one oxygen) across all La-doped compositions. The average V_2_O_7_ population, from ten runs per composition and temperature, peaks near *x* = 0.10 and remains appreciable at higher *x* values (Figure S14), thereby providing clear evidence for the vacancy-mediated transport mechanism assisted by VO_4_ rotations.

**Figure 8:**
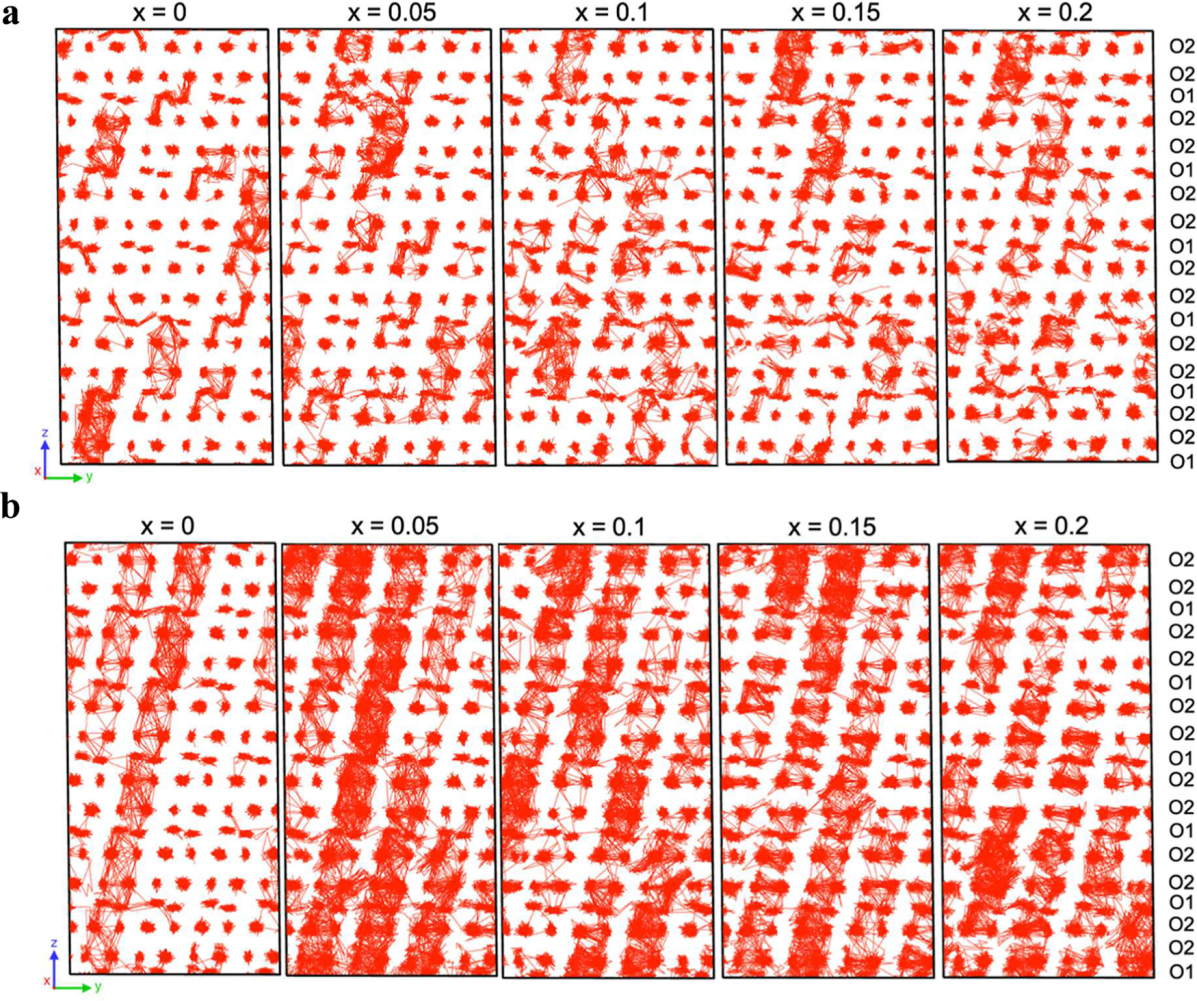
MTP-based MD trajectories of oxide-ion diffusion at (a) 700 K and (b) 1000 K for the Sr_3–3*x*_La_2*x*_V_2_O_8_ series. Metal cations are not shown for clarity.

MD simulations were also performed under wet air conditions to investigate proton transport in the Sr_3–3*x*_La_2*x*_V_2_O_8_·0.33H_2_O (*x* = 0–0.2) series. In contrast to the low activation energies for oxide-ion diffusion discussed above, the equivalent values for proton conductivity are significantly larger at 0.53–0.56 eV. This agrees well with our impedance findings, which show the suppression of proton transport in humid conditions. To understand why La doping results in such a significant decrease in proton transport, we calculated the binding energy of a proton at a Sr vacancy site in Sr_3–3*x*_La_2*x*_V_2_O_8_ (*x* = 0.1) using the following equation

1 $$E_{binding}=E_{V+H}-E_{bulk+H}$$

where *E*_V+H_ is the energy of the system with a proton in a vacancy site and *E*_bulk+H_ is the energy of the structure with the proton in a stable interstitial site far from the Sr vacancy. Using this approach, the binding energy was found to be −1.29 eV, indicating that the Sr vacancy acts as a strong trap site for protons, thereby resulting in proton localization at Sr vacancy sites and hindered long-range proton transport.

## Conclusion

Palmierite oxides are a versatile group of materials which can accommodate different doping strategies that can greatly enhance ionic conductivity. It has previously been shown that introducing oxygen interstitials into the palmierite structure improves the bulk ionic conductivity by 2 orders of magnitude. This is due to the change in the oxide-ion migration mechanism from the cog-wheel mechanism to an interstitialcy mechanism. Here, we have shown that by substituting La^3+^ for Sr^2+^ in Sr_3_V_2_O_8_, oxide-ion conduction via the cog-wheel mechanism can also be significantly improved. Results from neutron diffraction data and MD simulations shows that the V–O1 bond length becomes longer with increasing La concentration. This causes the oxygen situated at the O1 position to become more mobile making it easier for oxide ions to migrate to a vacant site. An increase in the distortion of the VO_4_ tetrahedra is also observed, which enhances dynamic lattice disorder and facilitates oxide ion migration. The oxide ion migration is further enhanced by the presence of significant cation vacancies at the Sr2 site so that the bulk oxide ion conductivity increases by an order of magnitude as *x* is increased from 0 to 0.15 in Sr_3–3*x*_La_2*x*_V_2_O_8_. MD simulations further show that La doping results in extended, interconnected trajectories and enhanced long-range oxide-ion diffusion. In contrast, our impedance and molecular dynamics analysis show suppressed proton conductivity upon La doping in Sr_3_V_2_O_8_ due to the presence of Sr vacancies. This was found to be the result of strong binding between protons and Sr vacancies, as revealed by DFT calculations.

## Experimental Section

Sr_3–3*x*_La_2*x*_V_2_O_8_ (*x* = 0.00, 0.05, 0.10, 0.15, 0.20, 0.25) samples were synthesized using the solid–state reaction method. Stoichiometric amounts of SrCO_3_ (>99.9% Sigma-Aldrich), V_2_O_5_ (99.95% Sigma-Aldrich), and La_2_O_3_ (99.95% Sigma-Aldrich) were ground, pressed into a pellet, heated at 1200 °C for 10 h, and then cooled to room temperature at 5 °C/min. The heating step was repeated until a phase pure product was obtained. The purity of the sample was confirmed using a PANalytical Empyrean diffractometer equipped with a Cu Kα tube and a Johansson monochromator. Data were recorded in the range 10° < 2θ < 120° with a step size of 0.013°.

The electrical properties of the Sr_3–3*x*_La_2*x*_V_2_O_8_ samples were measured by AC impedance spectroscopy with a Solartron 1260 analyzer in the frequency range 0.1 Hz-1 MHz with an alternating voltage of 0.1 V. Measurements were performed on platinum-coated dense pellets (∼90% of the theoretical density) by sintering samples (∼10 mm diameter, ∼1 mm thickness) for 2 h at 1200 °C. Data was collected upon cooling from 800 °C under a range of atmospheres in a sealed tube furnace, allowing 2 h of equilibration at each temperature step. For the measurements in air, O_2_ and N_2_ the employed gas was dried by flowing through a column of a commercial desiccant (Drierite) (*p*H_2_O < 10^–4^ atm). Humidified air was produced by bubbling air through a water-filled Dreschel bottle (*p*H_2_O ∼ 0.021 atm). The total resistivity (bulk and grain boundary) for each composition was extracted from the high-frequency intercept on the real impedance axis. Equivalent circuit analysis was then used to determine the individual bulk and grain boundary responses. More details can be found in Supporting Information.

Variable temperature time-of-fight (TOF) powder neutron diffraction data were collected on the POLARIS diffractometer at the ISIS Neutron and Muon Facility (Rutherford Appleton Laboratory, Harwell, Oxford, UK). Five g of sample were loaded into an 8 mm diameter thin-walled vanadium sample can and mounted in a furnace on the beamline. Diffraction data were collected at room temperature and at 700 °C for the Sr_3–3*x*_La_2*x*_V_2_O_8_ (*x* = 0.00, 0.05, 0.10 and 0.20) samples. For the *x* = 0.15 sample, additional data sets were also collected at intermediate temperatures of 200, 350, and 500 °C during heating. All data sets were collected for ∼2 h each (∼350 μAh integrated proton beam current to the ISIS target). Normalized diffraction patterns were analyzed by Rietveld structure refinement with the GSAS/EXPGUI software package.^21,22^ Minimum bounding ellipsoid analysis was performed using the PIEFACE software to evaluate the relaxation of the average metal polyhedral units upon increased lanthanum doping at temperature.^23^ The polyhedral distortion was quantified by the standard deviation, σ­(R), of three primary ellipsoid radii, R1, R2 and R3.

### Computational Methods

The Doped and the Site-Occupancy Disorder (SOD) packages were used to generate the La-doped Sr_3–3*x*_La_2*x*_V_2_O_8_ (*x* = 1/3) supercell.^24,25^ Density functional theory (DFT) calculations were carried out using the Vienna Ab initio Simulation Package (VASP).^26^ For static geometry optimizations, a 2 × 2 × 1 *k*-point grid and a plane-wave energy cutoff of 600 eV were employed. Ab initio molecular dynamics (AIMD) simulations were conducted in the *NVT* ensemble on a Sr_3–3*x*_La_2*x*_V_2_O_8_·0.33H_2_O (*x* = 1/3) supercell composed of 164 ions, using a reduced plane-wave cutoff of 450 eV and a Γ-point *k*-mesh. The simulations utilized a 1 fs time step and the Nosé–Hoover thermostat. Calculations were performed at three strain values (−0.05, 0 and 0.05) and at four temperatures (300, 600, 900, and 1200 K). For each temperature and strain condition, a 50 ps production run was carried out and snapshots were collected every 0.5 ps, yielding 100 configurations per condition and a total of 1200 structures for initial moment tensor potential (MTP) training.

MTP models were trained and evaluated using the MLIP-3 code, while classical molecular dynamics (MD) simulations were executed with LAMMPS package.^27–30^ Initially, a 16th-order MTP was pretrained using the AIMD-generated data set, incorporating 897 fitting parameters. After three rounds of passive learning and 18 rounds of active learning, a total of 5505 configurations were used in the potential training. The resulting model achieved root-mean-square errors of 2.9 meV/atom (energy), 0.28 eV/Å (forces) and 0.70 kBar (stresses) compared to the DFT data.

To investigate the diffusion behavior of Sr_3–3*x*_La_2*x*_V_2_O_8_, MD simulations within the *NVT* ensemble were performed using the trained MTP model on 4 × 4 × 2 supercells. A 1.3% vacancy concentration was introduced at oxygen sites to promote long-range transport and ten independent simulations were run for 10 ns at each temperature. The self-diffusion coefficients of oxide ions were determined from the mean-squared displacement (MSD)^31^ using

$$\left\langle r_{i}^{2}\left(t\right)\right\rangle =6D_{0}t$$

where ⟨*r*_*i*_^2^(*t*)⟩ is the MSD, *D*_0_ is the diffusion coefficient and *t* is time. The Nernst–Einstein equation was used to calculate the ionic conductivity σ of the system^32^

$$\sigma =\frac{e^{2}}{\kappa_{B}TV}\sum_{i}N_{i}q_{i}^{2}D_{0}$$

where *e* is the electric charge unit, κ_B_ is the Boltzmann constant, *T* is temperature, *q*_*i*_ is the charge of the ion and *N*_*i*_ is the number of oxide ions.

## Supplementary Material



## Notes

The Supporting Information document contains X-ray diffraction patterns, additional complex impedance plots, equivalent circuit models, Rietveld refinement figures, table of bond lengths and angles, tables of results from the PIEFACE analysis, a table of NEB-calculated energy barriers, and figures from MD simulations (V–O radial distribution functions and the average number of transient V_2_O_7_ dimers as a function of doping) (PDF)
