# Supplementary material for: Enhanced Oxide Ion Diffusion by Lanthanum Substitution in the Palmierite Sr3–3x La2x V2O8 via Increased Tetrahedral Distortion and Cation Vacancies
Source: Chem Mater. 2025 Sep 19;37(19):7987–95. doi: 10.1021/acs.chemmater.5c01856 (PMC12529909; doi:10.1021/acs.chemmater.5c01856)
Supplement: Supplementary file 1 [file cm5c01856_si_001.pdf]

Supplementary Information

for

Enhanced Oxide Ion Diffusion by Lanthanum Substitution in the Palmierite $Sr_{3-3x}La_{2x}V_2O_8$ via Increased Tetrahedral Distortion and Cation Vacancies

Victoria Watson[1], Ying Zhou[2], Ronald I. Smith[3], Sacha Fop[1], Yi Sun [4], Zongping Shao[4], San Ping Jiang[4, 5], Oscar J. B. Ballantyne[2], James A. Dawson[2] and Abbie C. Mclaughlin[1]*

[1] Advanced Centre for Energy and Sustainability (ACES), The Chemistry Department, University of Aberdeen, Aberdeen AB24 3UE, United Kingdom

[2]Chemistry − School of Natural and Environmental Sciences, Newcastle University, Newcastle, NE1 7RU, UK

[3] ISIS Neutron and Muon Source, Rutherford Appleton Laboratory, Chilton, Didcot, OX11 0QX, UK

[4] WA School of Mines: Minerals, Energy and Chemical Engineering, Curtin University, WA 6102, Australia

[5] Foshan Xianhu Laboratory of the Advanced Energy Science and Technology Guangdong Laboratory, Foshan 528216, China

* a.c.mclaughlin@abdn.ac.uk

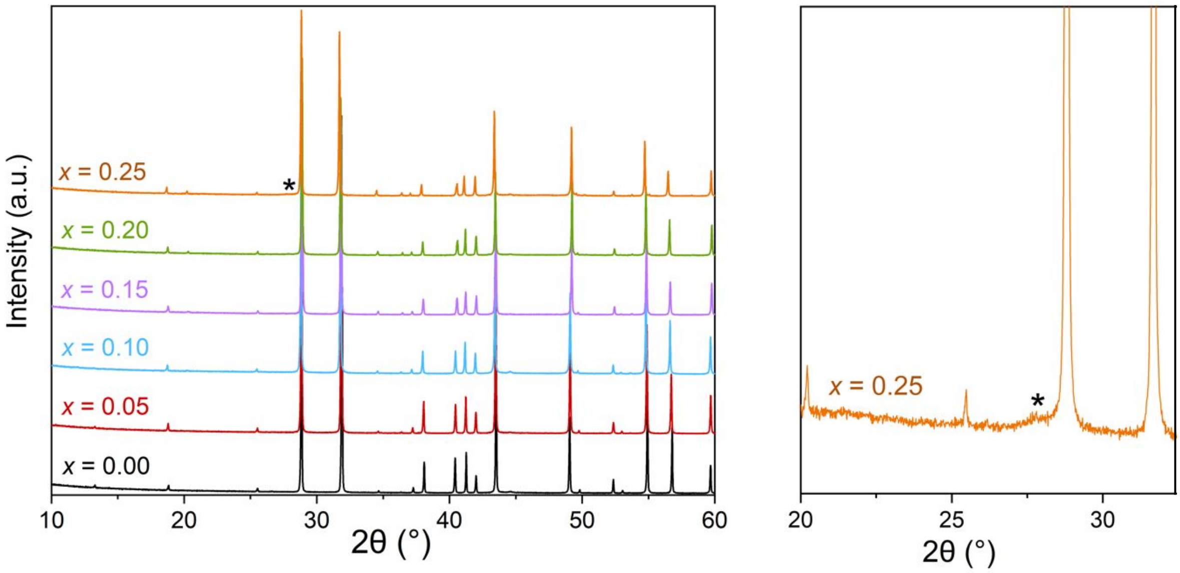

Figure S1. X-ray diffraction pattern of prepared samples $Sr_3V_2O_8$ (black), $Sr_{2.85}La_{0.10}V_2O_8$ (red), $Sr_{2.7}La_{0.20}V_2O_8$ (blue), $Sr_{2.55}$ $La_{0.30}V_2O_8$ (lilac), $Sr_{2.4}$ $La_{0.40}V_2O_8$ (green) and $Sr_{2.25}La_{0.\ 50}V_2O_8$ (orange). *indicates the presence of a La_2O_3 impurity. The right panel shows the LaO_3 impurity for the $Sr_{2.25}La_{0.\ 50}V_2O_8$ sample.

Equivalent Circuit Analysis

An equivalent circuit (EC) fitting procedure was employed to extract the individual bulk and grain boundary responses from the impedance data collected for the $Sr_{3-3x}La_{2x}V_2O_8$ series. The equivalent circuit depicted in Figure S2 was used to model the impedance data.

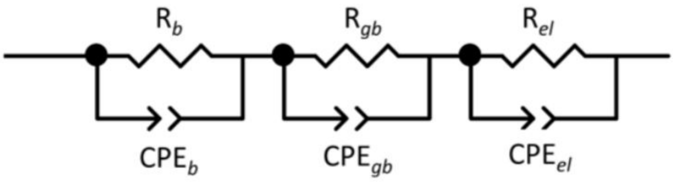

Figure S2. Equivalent circuit used to model the impedance data for the the $Sr_{3-3x}La_{2x}V_2O_8$ series recorded in dry air. R indicates a resistor, CPE is a constant phase element; the subscript b stands for bulk, gb for grain boundary and el for electrode.

The equivalent circuit in Figure S2 was employed to model the data for the $Sr_{3-3x}La_{2x}V_2O_8$ samples for all compositions (x = 0.0 – 0.20), in agreement with a previous report on $Sr_3V_2O_8$.[1] The complex impedance data of the $Sr_3V_2O_8$ sample is composed by an overlapping bulk and grain boundary and includes a Warburg signal in the low frequency region. The complex data of the $Sr_{3-3x}La_{2x}V_2O_8$ (x = 0.05, 0.1, 0.15,0.2,0.25) samples show two distinct and separate arcs which indicate the individual bulk and grain boundary responses. There is also a Warburg signal in the low frequency region. At higher temperatures >675 °C when the grain boundary was no longer visible the bulk conductivity was assumed to correspond with the total conductivity and was extracted from the high-frequency intercept of the electrode response on the real impedance axis.

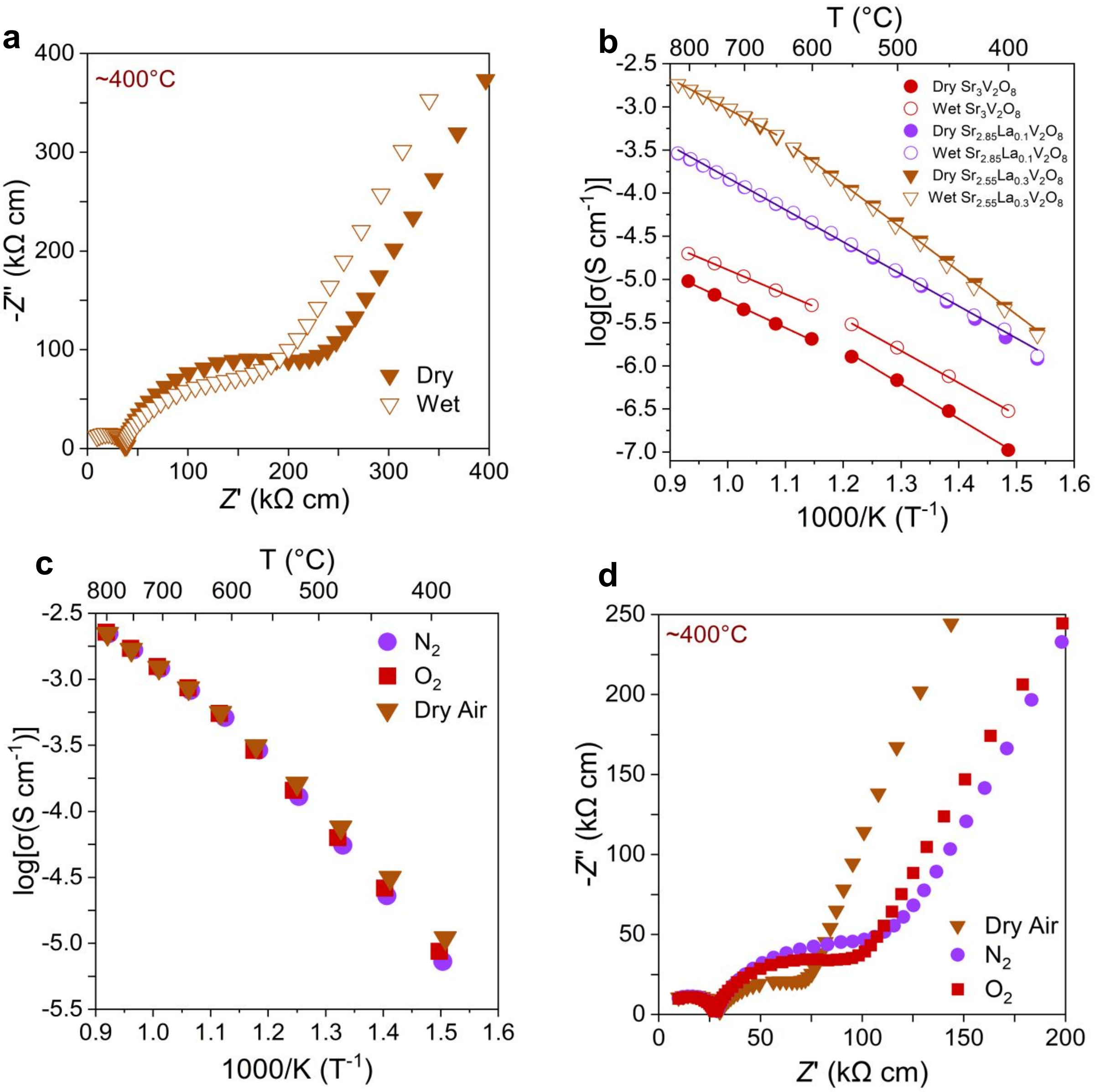

Figure S3. a) Complex impedance plot for the $Sr_{2.55}La_{0.3}V_2O_8$ sample at 400°C under dry and wet air. b) Arrhenius plot of the total conductivity of selected samples under dry and wet air indicating that proton conductivity is supressed with La doping. * Data obtained from ref 15. c) Arrhenius plot of the total conductivity of the $Sr_{2.55}La_{0.3}V_2O_8$ sample under dry air, O_2 and N_2. d) Complex impedance plot of the $Sr_{2.55}La_{0.3}V_2O_8$ sample at ~400 °C under dry air, O_2 and N_2. (need rest of impedance results).

The complex impedance plot for $Sr_{2.55}La_{0.3}V_2O_8$ at 400 °C under different atmospheres is shown in Figure S3d. The high-frequency arc associated with the bulk response show no change under O_2 and N_2 and is therefore independent of pO_2. The grain boundary resistance

shows a slight increase when measured under N_2 and O_2. A similar effect has previously been reported for NBT when measured under different gases.[2]

Stability of $Sr_{2.55}La_{0.3}V_2O_8$ under different atmospheres

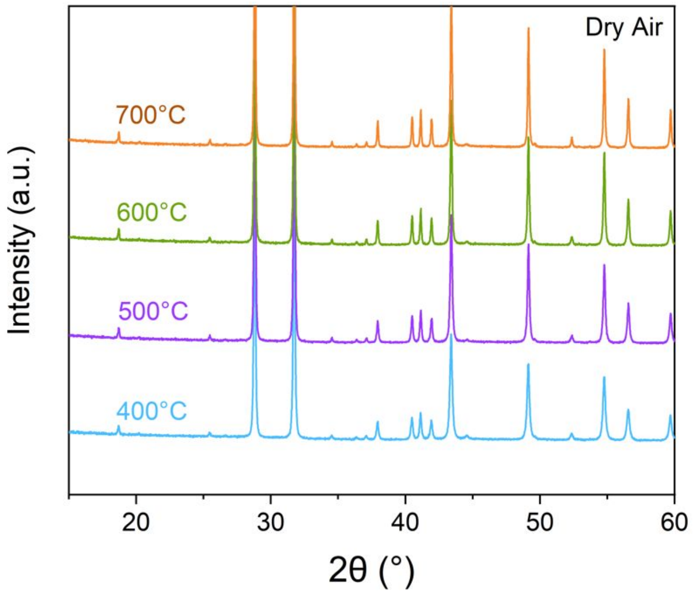

Figure S4. X-ray diffraction pattern of $Sr_{2.55}La_{0.3}V_2O_8$ under dry air at 400 °C (blue); 500°C purple); 600 °C (green); 700 °C (orange).

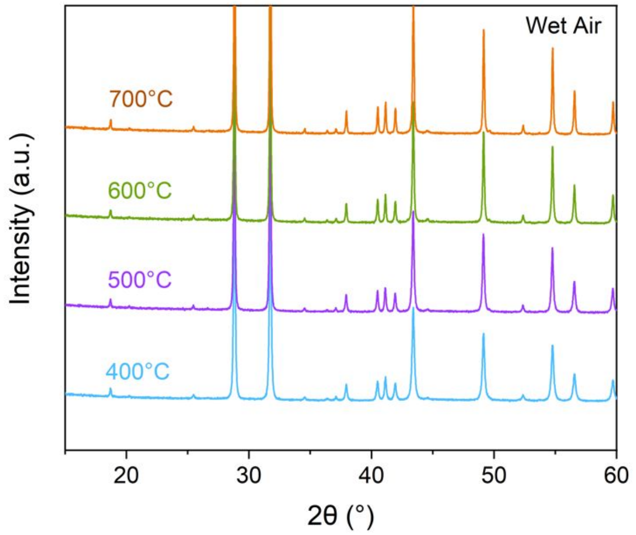

Figure S5. X-ray diffraction pattern of $Sr_{2.55}La_{0.3}V_2O_8$ under wet air at 400 °C (blue); 500 °C purple); 600 °C (green); 700 °C (orange).

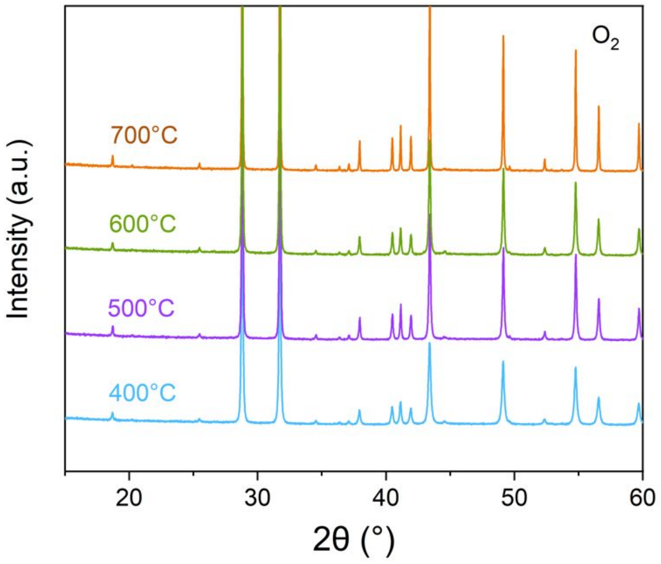

Figure S6. X-ray diffraction pattern of $Sr_{2.55}La_{0.3}V_2O_8$ under O_2 at 400 °C (blue); 500 °C purple); 600 °C (green); 700 °C (orange).

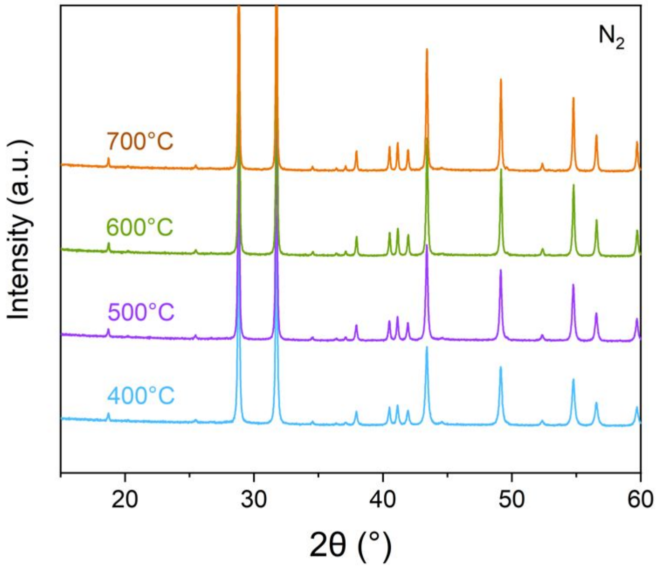

Figure S7. X-ray diffraction pattern of $Sr_{2.55}La_{0.3}V_2O_8$ under N_2 at 400 °C (blue); 500 °C purple); 600 °C (green); 700 °C (orange).

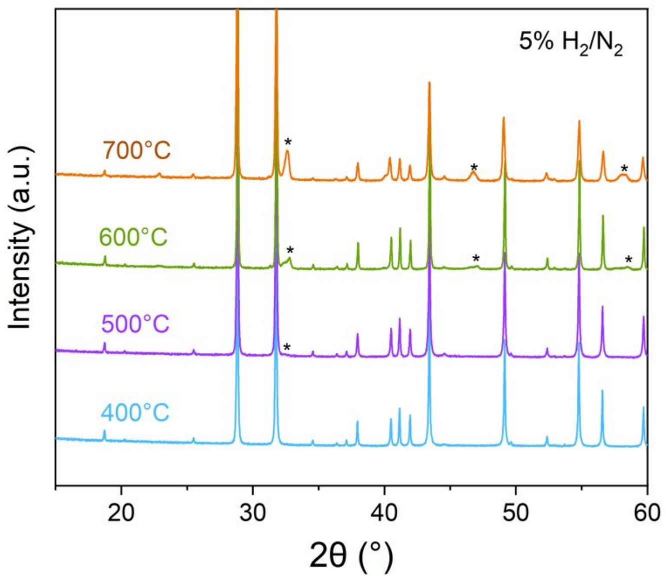

Figure S8. X-ray diffraction pattern of $Sr_{2.55}La_{0.3}V_2O_8$ under 5% H_2/N_2 at 400 °C (blue); 500 °C purple); 600 °C (green); 700 °C (orange). The impurity phase (*) was determined to be $La_{0.5}Sr_{0.5}VO_{2.95}$.

Figures S4-S7 show no evidence of additional peaks indicating that the $Sr_{2.55}La_{0.3}V_2O_8$ phase is stable under dry/humidified air, O_2 and N_2. However, Figure S8 does show evidence of large additional peaks attributed to an impurity $La_{0.5}Sr_{0.5}VO_{2.95}$ phase forming at ~32.5°, ~46.5° and ~58° at temperatures from 500 °C -700 °C indicating that $Sr_{2.55}La_{0.3}V_2O_8$ is not stable under 5% H_2/N_2. This suggests that the introduction of lanthanum into the structure causes it to become unstable under reducing conditions. This is surprising as the parent compound, $Sr_3V_2O_8$, was found to be stable under a range of atmospheres including 5% H_2/N_2.

Table S1. Refined atomic parameters and agreement factors for $Sr_{3-3x}La_{2x}V_2O_8$ (x = 0.00 - 0.20) from the Rietveld fit to the $R\overline{3}m$ model from X-ray diffraction data collected on the PANalytical Empyrean diffractometer room temperature.

		***x* = 0.00**	***x* = 0.05**	***x* = 0.10**	***x* = 0.15**	***x* = 0.20**
**Sr1**	Occupancy	1	0.911(7)	0.761(7)	0.650(7)	0.550(6)
(0 0 0) 3*a*	U_{iso}	0.0182(5)	0.0210(11)	0.0188(14)	0.0209(12)	0.0345(12)
**La1**	Occupancy	0	0.1	0.2	0.3	0.4
(0 0 0) 3*a*	U_{iso}	-	0.0210(11)	0.0188(14)	0.0209(12)	0.0345(12)
**Sr2**	Occupancy	1	0.969(3)	0.969(3)	0.950(4)	0.925(3)
(0 0 *z*) 6*c*	U_{iso}	0.0085(4)	0.0073(7)	0.0101(10)	0.0146(10)	0.0100(10)
	z	0.79760(7)	0.79667(11)	0.79827(12)	0.79858(11)	0.79845(10)
**V1**	Occupancy	1	1	1	1	1
(0 0 *z*) 6*c*	U_{iso}	0.0076(7)	0.0073(10)	0.0059(13)	0.0089(10)	0.0066(8)
	z	0.5954(1)	0.5956(2)	0.5967(3)	0.5967(2)	0.5987(2)
**O1**	Occupancy	1	1	1	1	1
(0 0 *z*) 6*c*	U_{iso}	0.0173(22)	0.0210(40)	0.0274(60)	0.0250(45)	0.0139(36)
	z	0.68277(54)	0.68275(80)	0.68163(102)	0.68572(89)	0.68820(75)
**O2**	Occupancy	1	1	1	1	1
(*x y z*) 18*h*	U_{iso}	0.0161(12)	0.0171(20)	0.0274(47)	0.0130(17)	0.0495(26)
	x	0.49333(39)	0.48863(58)	0.49240(67)	0.49558(67)	0.48846(67)
	y	0.50666(39)	0.51137(58)	0.50760(67)	0.50441(67)	0.51154(67)
	z	0.23225(23)	0.23082(38)	0.23500(37)	0.23466(29)	0.23042(43)
χ^2		2.810	4.356	3.956	3.779	3.650
R_p**(%)**		4.58	5.08	7.26	4.96	4.93
R_{wp}**(%)**		6.13	7.24	10.34	7.08	7.02

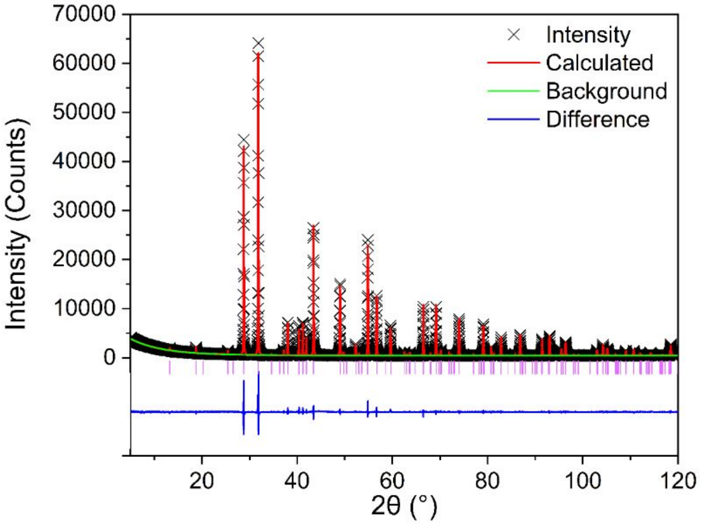

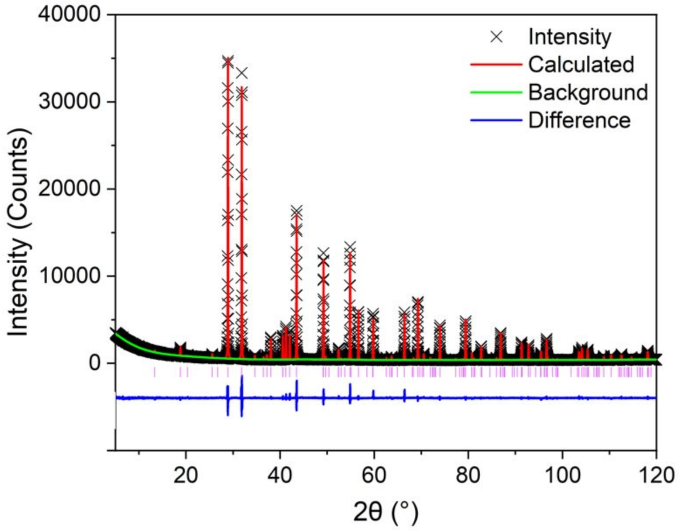

Figure S9. Rietveld refinement fits to the $R\overline{3}m$ *H* model from X-ray diffraction data for $Sr_3V_2O_8$ (top) and $Sr_{2.55}La_{0.3}V_2O_8$ (bottom). Black crosses show the observed data, the red line is the Rietveld fit and the green line is the background function, the blue line shows the difference between the calculated and observed data. Vertical pink lines show the reflection positions for $Sr_3V_2O_8$ and $Sr_{2.55}La_{0.3}V_2O_8$ respectively.

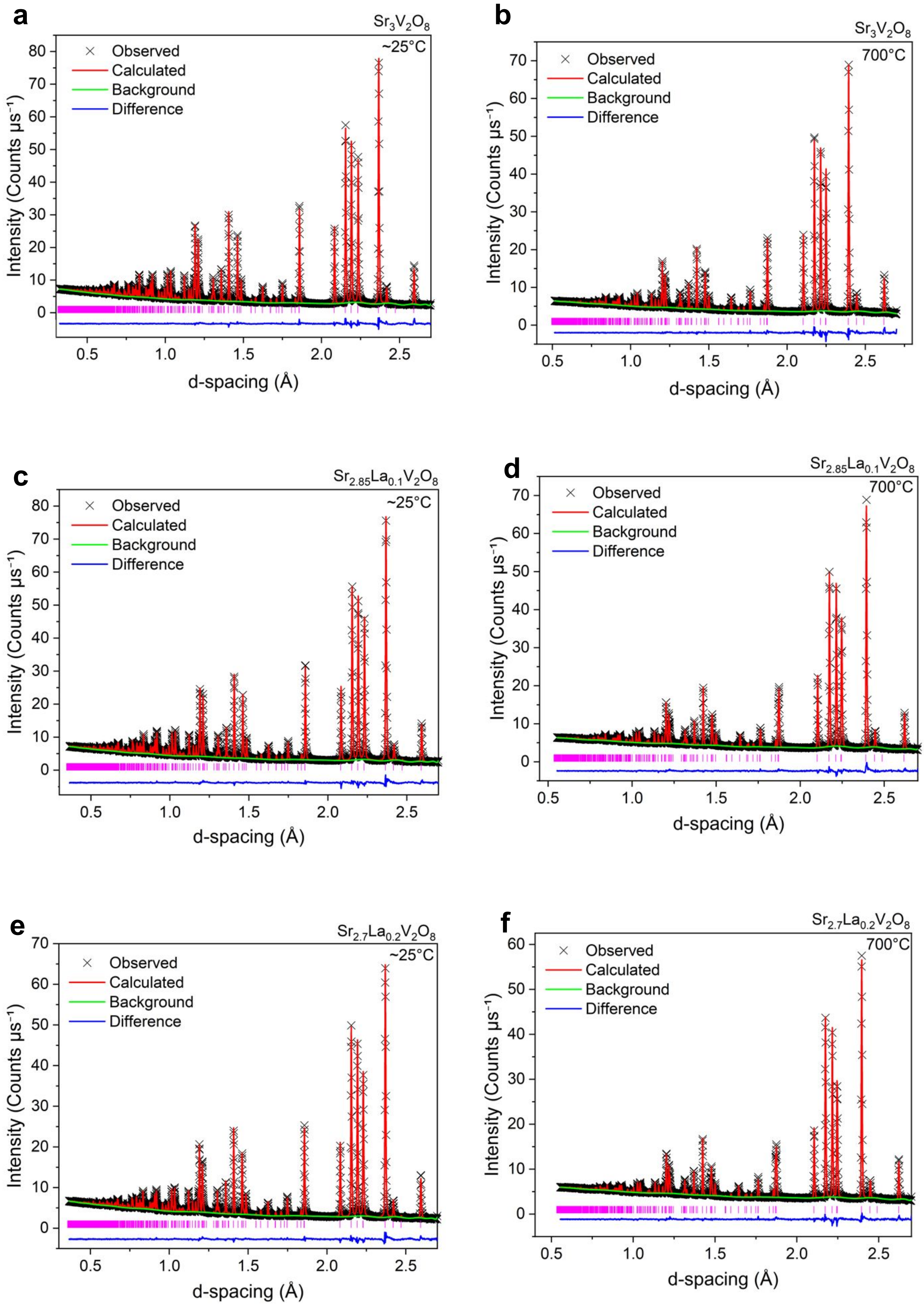

a
$Sr_3V_2O_8$
~25°C
b
$Sr_3V_2O_8$
700°C
c
$Sr_{2.85}La_{0.1}V_2O_8$
~25°C
d
$Sr_{2.85}La_{0.1}V_2O_8$
700°C
e
$Sr_{2.7}La_{0.2}V_2O_8$
~25°C
f
$Sr_{2.7}La_{0.2}V_2O_8$
700°C
Observed
Calculated
Background
Difference
Intensity (Counts μs^{-1})
d-spacing (Å)

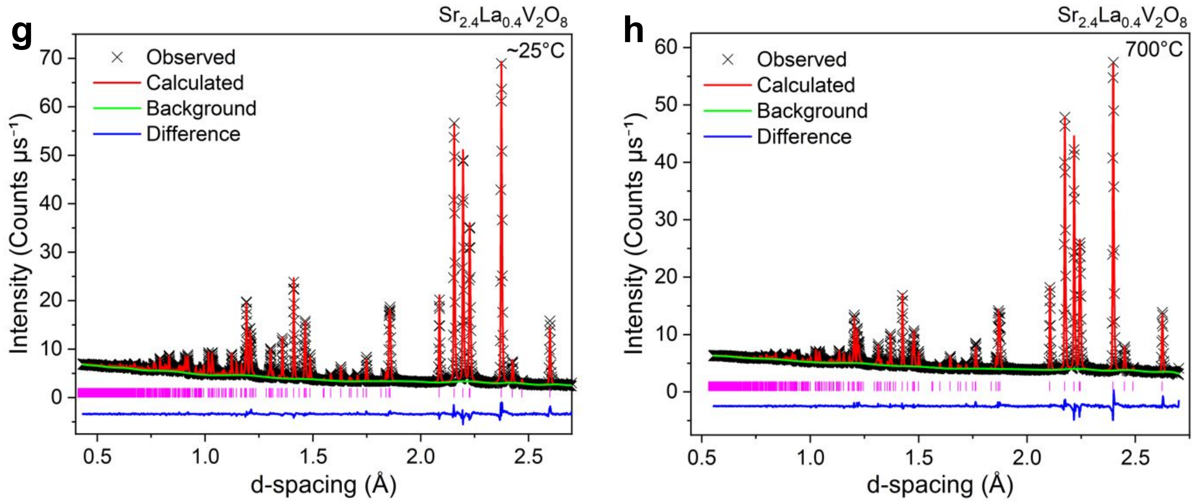

Figure S10. Rietveld refinement fits to the $R\overline{3}m$ model for a) $Sr_3V_2O_8$ at 25°C b) $Sr_3V_2O_8$ at 700°C. c) $Sr_{2.85}La_{0.1}V_2O_8$ at 25°C. d) $Sr_{2.85}La_{0.1}V_2O_8$ at 700°C. e) $Sr_{2.70}La_{0.2}V_2O_8$ at 25°C. f) $Sr_{2.70}La_{0.2}V_2O_8$ at 700°C. g) $Sr_{2.40}La_{0.4}V_2O_8$ at 25°C. h) $Sr_{2.40}La_{0.4}V_2O_8$ at 700°C. Black crosses show the observed data, the red line is the Rietveld fit and the green line is the background function, the blue line shows the difference between the calculated and observed data. Vertical pink lines show the reflection positions for each sample.

Table S2. Refined atomic parameters, cell parameters and agreement factors for $Sr_{3-3x}La_{2x}V_2O_8$ (x = 0.00 - 0.20) from the Rietveld fit to the $R\overline{3}m$ model from neutron diffraction data collected on the POLARIS diffractometer at 25°C. A fixed isotropic displacement parameter, Uiso, obtained from initial refinements of the room temperature XRD data was employed for V.

25 °C		***x* = 0.00**	***x* = 0.05**	***x* = 0.10**	***x* = 0.15**	***x* = 0.20**
***a* (Å)**		5.61632(5)	5.61879(3)	5.62747(6)	5.62886(6)	5.64013(8)
***c* (Å)**		20.0928(2)	20.0564(2)	20.0480(2)	20.0187(2)	20.0232(3)
***V* (Å³)**		548.877(11)	548.366(13)	549.828(14)	549.297(16)	551.629(19)
		***x* = 0.00**	***x* = 0.05**	***x* = 0.10**	***x* = 0.15**	***x* = 0.20**
**Sr1**	Occupancy	1	0.896(2)	0.801(2)	0.699(2)	0.597(3)
(0 0 0) 3*a*	U_{11}=U_{22}	0.0239(2)	0.0174(2)	0.0202(2)	0.0228(3)	0.0265(3)
	U_{33}	0.0053(2)	0.0174(2)	0.0202(2)	0.0228(3)	0.0265(3)
	U_{12}	0.0120(1)	0.0087(1)	0.0101(1)	0.0114(1)	0.0132(2)
**La1**	Occupancy	0	0.1	0.2	0.3	0.4
(0 0 0) 3*a*	U_{11}=U_{22}	-	0.0174(2)	0.0202(2)	0.0228(3)	0.0265(3)
	U_{33}	-	0.0174(2)	0.0202(2)	0.0228(3)	0.0265(3)
	U_{12}	-	0.0087(1)	0.0101(1)	0.0114(1)	0.0132(2)
**Sr2**	Occupancy	1	0.977(1)	0.950(1)	0.925(1)	0.902(1)
(0 0 *z*) 6*c*	U_{11}=U_{22}	0.0129(1)	0.0168(2)	0.0212(2)	0.0272(3)	0.0358(3)
	U_{33}	0.0052(2)	0.0043(2)	0.0048(2)	0.0046(3)	0.0046(3)
	U_{12}	0.0065(1)	0.0084(1)	0.0106(1)	0.0136(1)	0.0179(2)
	z	0.79784(2)	0.79821(2)	0.79869(3)	0.79913(3)	0.79973(3)
**V1**	Occupancy	1	1	1	1	1
(0 0 *z*) 6*c*	U_{iso}	0.0076	0.0073	0.0059	0.0089	0.0164
	z	0.5962(3)	0.5960(4)	0.5949(4)	0.5939(4)	0.5949(4)
**O1**	Occupancy	1	1	1	1	1
(0 0 *z*) 6*c*	U_{11}=U_{22}	0.0341(2)	0.0419(3)	0.0490(3)	0.0561(3)	0.0674(4)
	U_{33}	0.0041(2)	0.0039(3)	0.0047(3)	0.0042(3)	0.0041(4)
	U_{12}	0.0171(1)	0.0210(1)	0.0245(2)	0.0280(2)	0.0337(2)
	z	0.67755(3)	0.67797(3)	0.67848(3)	0.67878(4)	0.67922(4)
**O2**	Occupancy	1	1	1	1	1
(*x y z*) 18*h*	U_{11}=U_{22}	0.0248(1)	0.0249(1)	0.0264(1)	0.0287(2)	0.0326(2)
	U_{33}	0.0124(1)	0.0138(2)	0.0160(2)	0.0180(2)	0.0212(2)
	U_{12}	0.0210(1)	0.0210(2)	0.0223(2)	0.0243(2)	0.0275(2)
	U_{13} = -U_{23}	0.0014(1)	0.0011(1)	0.0011(1)	0.0009(1)	0.0007(1)
	x	0.49908(4)	0.49886(5)	0.49866(5)	0.49849(5)	0.49819(6)
	y	0.50092(4)	0.50114(5)	0.50134(5)	0.50152(5)	0.50181(6)
	z	0.23187(2)	0.23232(2)	0.23294(2)	0.23338(2)	0.23394(2)
χ^2		6.137	6.736	5.649	6.952	8.187
R_p(%)		1.96	1.96	1.90	2.06	2.07
R_{wp}(%)		1.03	1.16	1.12	1.24	1.30

Table S3. Refined atomic parameters, cell parameters and agreement factors for $Sr_{3-3x}La_{2x}V_2O_8$ (x = 0.00 - 0.20) from the Rietveld fit to the $R\bar{3}m$ model from neutron diffraction data collected on the POLARIS diffractometer at 700°C. A fixed isotropic displacement parameter, Uiso, obtained from initial refinements of the room temperature XRD data was employed for V.

700 °C		x = 0.00	x = 0.05	x = 0.10	x = 0.15	x = 0.20
***a* (Å)**		5.68315(6)	5.68168(7)	5.69180(6)	5.68871(8)	5.69711(7)
***c* (Å)**		20.2121(2)	20.1800(3)	20.1896(2)	20.1586(3)	20.1695(3)
**V ($Å^3$)**		565.353(16)	564.162(19)	566.447(15)	564.963(21)	566.940(17)
		x = 0.00	x = 0.05	x = 0.10	x = 0.15	x = 0.20
**Sr1**	Occupancy	1	0.896	0.801	0.699	0.597(3)
(0 0 0) 3*a*	U_{11}=U_{22}	0.0502(4)	0.0362(3)	0.0393(3)	0.0408(3)	0.0420(3)
	U_{33}	0.0194(4)	0.0362(3)	0.0393(3)	0.0408(3)	0.0420(3)
	U_{12}	0.0251(2)	0.0181(1)	0.0197(2)	0.0204(1)	0.0210(2)
**La1**	Occupancy	0	0.1	0.2	0.3	0.4
(0 0 0) 3*a*	U_{11}=U_{22}	-	0.0362(3)	0.0393(3)	0.0408(3)	0.0420(3)
	U_{33}	-	0.0362(3)	0.0393(3)	0.0408(3)	0.0420(3)
	U_{12}	-	0.0181(1)	0.0197(2)	0.0204(1)	0.0210(2)
**Sr2**	Occupancy	1	0.977	0.950	0.925	0.902
(0 0 *z*) 6*c*	U_{11}=U_{22}	0.0369(3)	0.0408(3)	0.0464(4)	0.0534(4)	0.0591(4)
	U_{33}	0.0185(3)	0.0153(3)	0.0139(4)	0.0140(4)	0.0142(5)
	U_{12}	0.0184(1)	0.0204(2)	0.0232(2)	0.0267(2)	0.0296(2)
	z	0.79729(3)	0.79766(3)	0.79818(3)	0.79868(3)	0.79923(4)
**V1**	Occupancy	1	1	1	1	1
(0 0 *z*) 6*c*	U_{iso}	0.0076	0.0073	0.0059	0.0089	0.0164
	z	0.5971(3)	0.5951(4)	0.5936(4)	0.5945(4)	0.5934(5)
**O1**	Occupancy	1	1	1	1	1
(0 0 *z*) 6*c*	U_{11}=U_{22}	0.0845(4)	0.0929(5)	0.0983(5)	0.1024(5)	0.1068(6)
	U_{33}	0.0157(5)	0.0162(5)	0.0165(6)	0.0182(6)	0.0170(7)
	U_{12}	0.0422(2)	0.0464(2)	0.0491(3)	0.0512(3)	0.0534(3)
	z	0.67661(4)	0.67704(4)	0.67729(5)	0.67780(5)	0.67823(5)
**O2**	Occupancy	1	1	1	1	1
(*x y z*) 18*h*	U_{11}=U_{22}	0.0536(2)	0.0533(2)	0.0535(2)	0.0563(3)	0.0571(3)
	U_{33}	0.0344(3)	0.0342(3)	0.0381(3)	0.0405(3)	0.0418(4)
	U_{12}	0.0423(2)	0.0431(3)	0.0436(3)	0.0453(3)	0.0463(3)
	U_{13} = -U_{23}	0.0020(1)	0.0021(1)	0.0019(1)	0.0007(1)	0.0006(2)
	x	0.49736(5)	0.49758(6)	0.49749(7)	0.49718(7)	0.49714(8)
	y	0.50264(5)	0.50242(6)	0.50251(7)	0.50282(7)	0.50285(8)
	z	0.23249 (2)	0.23309(3)	0.23362(3)	0.23407(3)	0.23456(3)
χ^2		3.629	4.291	3.927	4.064	4.406
R_p(%)		0.88	1.00	0.99	1.06	1.16
R_{wp}(%)		1.74	1.64	1.62	1.69	1.91

Table S4. Refined atomic parameters, cell parameters and agreement factors for $Sr_{2.55}La_{0.3}V_2O_8$ from the Rietveld fit to the $R\overline{3}m$ model from neutron diffraction data collected on the POLARIS diffractometer at 25 °C, 200 °C, 350 °C, 500 °C and 700 °C. A fixed isotropic displacement parameter, Uiso, obtained from initial refinements of the room temperature XRD data was employed for V.

$Sr_{2.55}La_{0.3}V_2O_8$		25 °C	200 °C	350 °C	500 °C	700 °C
a **(Å)**		5.62886(6)	5.64366(7)	5.65661(7)	5.6700(1)	5.68871(8)
c **(Å)**		20.0187(2)	20.0491(3)	20.0795(3)	20.1105(3)	20.1586(3)
**V (Å³)**		549.297(16)	553.028(17)	556.412(18)	559.913(19)	564.963(21)
		**25 °C**	**200 °C**	**350 °C**	**500 °C**	**700 °C**
**Sr1**	Occupancy	0.699(2)	0.699	0.699	0.699	0.699
(0 0 0) 3*a*	U_{11}=U_{22}	0.0228(3)	0.0265(2)	0.0305(2)	0.0350(3)	0.0408(3)
	U_{33}	0.0228(3)	0.0265(2)	0.0305(2)	0.0350(3)	0.0408(3)
	U_{12}	0.0114(1)	0.0133(1)	0.0152(1)	0.0175(1)	0.0204(1)
**La1**	Occupancy	0.3	0.3	0.3	0.3	0.3
(0 0 0) 3*a*	U_{11}=U_{22}	0.0228(3)	0.0265(2)	0.0305(2)	0.0350(3)	0.0408(3)
	U_{33}	0.0228(3)	0.0265(2)	0.0305(2)	0.0350(3)	0.0408(3)
	U_{12}	0.0114(1)	0.0133(1)	0.0152(1)	0.0175(1)	0.0204(1)
**Sr2**	Occupancy	0.925(1)	0.925	0.925	0.925	0.925
(0 0 *z*) 6*c*	U_{11}=U_{22}	0.0272(3)	0.0338(3)	0.0392(3)	0.0451(3)	0.0534(4)
	U_{33}	0.0046(3)	0.0071(3)	0.0091(3)	0.0093(4)	0.0140(4)
	U_{12}	0.0136(1)	0.0169(1)	0.0197(2)	0.0226(2)	0.0267(2)
	z	0.79913(3)	0.79911(3)	0.79897(3)	0.79886(3)	0.79868(3)
**V1**	Occupancy	1	1	1	1	1
(0 0 *z*) 6*c*	U_{iso}	0.0089	0.0089	0.0089	0.0089	0.0089
	Z	0.5939(4)	0.5955(4)	0.5955(4)	0.5953(4)	0.5945(4)
**O1**	Occupancy	1	1	1	1	1
(0 0 *z*) 6*c*	U_{11}=U_{22}	0.0561(3)	0.0687(4)	0.0773(4)	0.0868(5)	0.1024(5)
	U_{33}	0.0042(3)	0.0072(4)	0.0116(5)	0.0130(5)	0.0182(6)
	U_{12}	0.0280(2)	0.0343(2)	0.0386(2)	0.0434(2)	0.0512(3)
	Z	0.67878(4)	0.67868(4)	0.67836(4)	0.67813(4)	0.67780(5)
**O2**	Occupancy	1	1	1	1	1
(*x y z*) 18*h*	U_{11}=U_{22}	0.0287(2)	0.0346(2)	0.0407(2)	0.0455(2)	0.0562(3)
	U_{33}	0.0180(2)	0.0234(2)	0.0271(3)	0.0328(3)	0.0405(3)
	U_{12}	0.0243(2)	0.0287(2)	0.0333(2)	0.0372(3)	0.0453(3)
	U_{13} = -U_{23}	0.0009(1)	0.0010(1)	0.0014(1)	0.0011(1)	0.0065(1)
	X	0.49849(5)	0.49813(6)	0.49814(6)	0.49769(6)	0.49718(7)
	Y	0.50152(5)	0.50187(6)	0.50186(6)	0.50231(6)	0.50282(7)
	Z	0.23338(2)	0.23353(2)	0.23371(2)	0.23381(3)	0.23407(3)
χ^2		6.952	6.600	5.881	4.999	4.064
R_p(%)		2.06	1.13	1.18	1.11	1.06
R_{wp}(%)		1.24	1.88	1.92	1.77	1.69

Table S5. Selected bond lengths and angles for $Sr_{3-3x}La_{2x}V_2O_8$ (x = 0.00 - 0.20) from the Rietveld fit to the $R\bar{3}m$ model from neutron diffraction data at 25 °C.

**Bond Distance (Å)**	***x* = 0.00**	***x* = 0.05**	***x* = 0.10**	***x* = 0.15**	***x* = 0.20**
Sr1-O2	2.6104(4)	2.6023(4)	2.5947(4)	2.5869(5)	2.5825(5)
Sr2-O1	2.4171(7)	2.4116(8)	2.4096(9)	2.4090(9)	2.4124(10)
V-O1	1.634(6)	1.643(7)	1.676(7)	1.699(8)	1.689(8)
V-O2	1.7286(23)	1.7224(26)	1.7105(25)	1.6995(27)	1.7026(28)
**Bond Angle (°)**	***x* = 0.00**	***x* = 0.05**	***x* = 0.10**	***x* = 0.15**	***x* = 0.20**
O1-V-O2	111.14(19)	110.73(22)	109.59(23)	108.66(27)	108.93(27)
O2-V-O2	107.76(20)	108.19(24)	109.35(23)	110.27(26)	110.00(26)

Table S6. Selected bond lengths and angles for $Sr_{3-3x}La_{2x}V_2O_8$ (x = 0.00 - 0.20) from the Rietveld fit to the $R\bar{3}m$ model from neutron diffraction data at 700 °C.

**Bond Distance (Å)**	***x* = 0.00**	***x* = 0.05**	***x* = 0.10**	***x* = 0.15**	***x* = 0.20**
Sr1-O2	2.6329(5)	2.6194(6)	2.6144(6)	2.6064(6)	2.6017(7)
Sr2-O1	2.4393(9)	2.4341(10)	2.4408(11)	2.4368(11)	2.4411(12)
V-O1	1.607(7)	1.653(8)	1.690(8)	1.676(8)	1.708(10)
V-O2	1.7339(25)	1.7170(28)	1.7052(27)	1.7052(28)	1.6971(32)
**Bond Angle (°)**	***x* = 0.00**	***x* = 0.05**	***x* = 0.10**	***x* = 0.15**	***x* = 0.20**
O1-V-O2	111.37(21)	109.72(25)	108.37(26)	108.78(27)	107.74(33)
O2-V-O2	107.50(22)	109.22(25)	110.55(25)	110.15(26)	111.14(31)

Table S7. Selected bond lengths and angles for $Sr_{2.55}La_{0.3}V_2O_8$ from the Rietveld fit to the $R\overline{3}m$ model from neutron diffraction data at 25 °C, 200 °C, 350 °C, 500 °C and 700 °C.

Bond Distance (Å)	30 °C	200 °C	350 °C	500 °C	700 °C
Sr1-O2	2.5869(5)	2.5919(5)	2.5938(5)	2.6000(6)	2.6064(6)
Sr2-O1	2.4090(9)	2.4145(9)	2.4217(10)	2.4278(10)	2.4368(11)
V-O1	1.699(8)	1.668(8)	1.662(9)	1.665(8)	1.676(8)
V-O2	1.6995(27)	1.7102(29)	1.7135(30)	1.7107(28)	1.7052(28)
**Bond Angle (°)**	**30 °C**	**200 °C**	**350 °C**	**500 °C**	**700 °C**
O1-V-O2	110.437(15)	109.62(26)	109.55(27)	109.35(27)	108.78(27)
O2-V-O2	108.488(15)	109.33(27)	109.39(28)	109.59(26)	110.15(26)

Table S8. Minimum bounding ellipsoid data calculated by the PIEFACE software package for VO_4 tetrahedra at 25 °C. *R*1, *R*2 and *R*3 are the principal ellipsoid radii, <*R*> is the mean radius, σ(R) is a measure of the polyhedral distortion, *S* is the ellipsoidal shape parameter and *d* is the out-of-centre cation displacement.

Composition	*R1* (Å)	*R2* (Å)	*R3* (Å)	*<R>* (Å)	*σ(R)* (Å)	*S*	*d*
$Sr_3V_2O_8$	1.71015	1.71015	1.69302	1.70444	0.008076	-0.010017	0.058469
$Sr_{2.85}La_{0.1}V_2O_8$	1.70957	1.70957	1.68952	1.70288	0.009452	-0.011728	0.046274
$Sr_{2.7}La_{0.2}V_2O_8$	1.70920	1.70920	1.68714	1.70185	0.010399	-0.012905	0.011086
$Sr_{2.55}La_{0.3}V_2O_8$	1.70776	1.70776	1.68286	1.69946	0.011738	-0.014579	0.017115
$Sr_{2.4}La_{0.4}V_2O_8$	1.70818	1.70817	1.68083	1.69906	0.012891	-0.016008	0.007908

Table S9. Minimum bounding ellipsoid data calculated by the PIEFACE software package for VO_4 tetrahedra at 700 °C. *R*1, *R*2 and *R*3 are the principal ellipsoid radii, <*R*> is the mean radius, σ(R) is a measure of the polyhedral distortion, *S* is the ellipsoidal shape parameter and *d* is the out-of-centre cation displacement.

Composition	*R1* (Å)	*R2* (Å)	*R3* (Å)	<*R*> (Å)	σ*(R)* (Å)	*S*	*d*
$Sr_3V_2O_8$	1.71254	1.71254	1.67944	1.70150	0.015246	-0.019327	0.072353
$Sr_{2.85}La_{0.1}V_2O_8$	1.71434	1.71434	1.67418	1.70095	0.018933	-0.023427	0.021238
$Sr_{2.7}La_{0.2}V_2O_8$	1.71651	1.71651	1.67088	1.70130	0.021508	-0.026580	0.020390
$Sr_{2.55}La_{0.3}V_2O_8$	1.71233	1.71233	1.66907	1.69791	0.020394	-0.025264	0.009524
$Sr_{2.4}La_{0.4}V_2O_8$	1.714494	1.714493	1.66860	1.69920	0.021633	-0.026765	0.042960

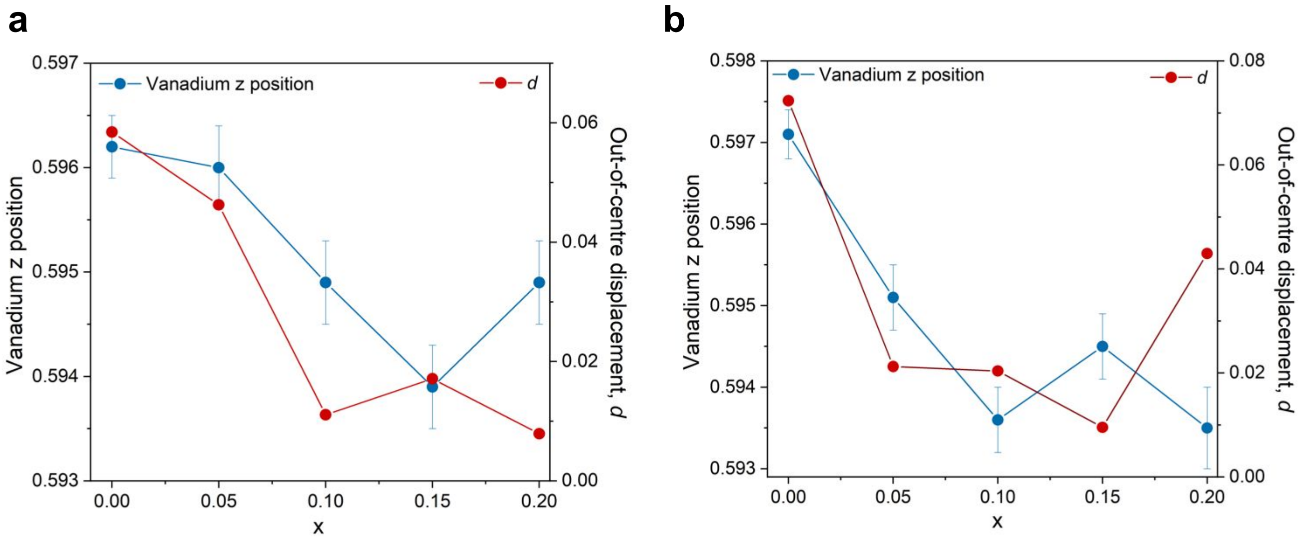

Figure S11. Variation of the vanadium *z* position and out-of-centre displacement, *d*, with increasing lanthanum content at (a) 25 and (b) 700 °C.

Table S10. Minimum bounding ellipsoid data calculated by the PIEFACE software package for VO_4 tetrahedra at selected temperatures for $Sr_{2.55}La_{0.3}V_2O_8$. *R*1, *R*2 and *R*3 are the principal ellipsoid radii, <*R*> is the mean radius, σ(R) is a measure of the polyhedral distortion, *S* is the ellipsoidal shape parameter and *d* is the out-of-centre cation displacement.

T (°C)	*R1* (Å)	*R2* (Å)	*R3* (Å)	<*R*> (Å)	σ*(R)* (Å)	*S*	*d*
30	1.7078	1.70776	1.68286	1.69946	0.011738	-0.014579	0.017115
200	1.72159	1.70737	1.68212	1.70369	0.016322	-0.006535	0.014033
350	1.71254	1.7125	1.67699	1.70069	0.016759	-0.020759	0.012197
500	1.71212	1.71212	1.67370	1.69931	0.018111	-0.022439	0.008546
700	1.71233	1.71233	1.66907	1.69791	0.020394	-0.025264	0.009524

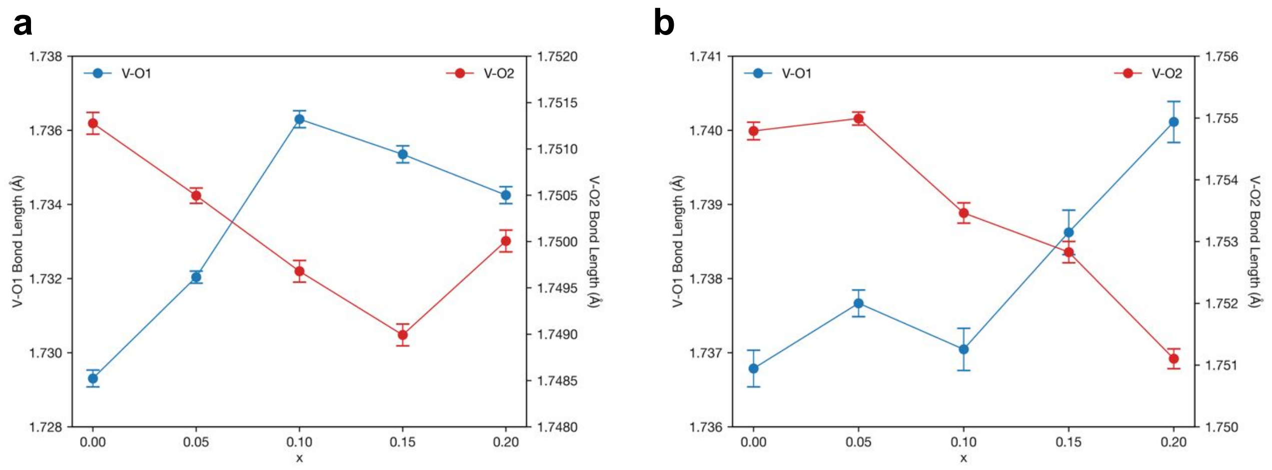

Figure S12. Variation of V-O1 and V-O2 bond lengths with increasing La content at (a) 700 and (b) 1000 K predicted by MD simulations.

Table S11. NEB-calculated energy barriers for O1–O1, O1–O2, and O2–O2 hopping at 0 K with increasing lanthanum concentration.

Composition	O1-O1 (eV)	O1-O2 (eV)	O2-O2 (eV)
$Sr_3V_2O_8$	1.244	0.497	0.818
$Sr_{2.85}La_{0.1}V_2O_8$	1.097	0.353	0.506
$Sr_{2.7}La_{0.2}V_2O_8$	0.740	0.499	0.269
$Sr_{2.55}La_{0.3}V_2O_8$	0.765	0.477	0.281
$Sr_{2.4}La_{0.4}V_2O_8$	0.709	0.657	0.330

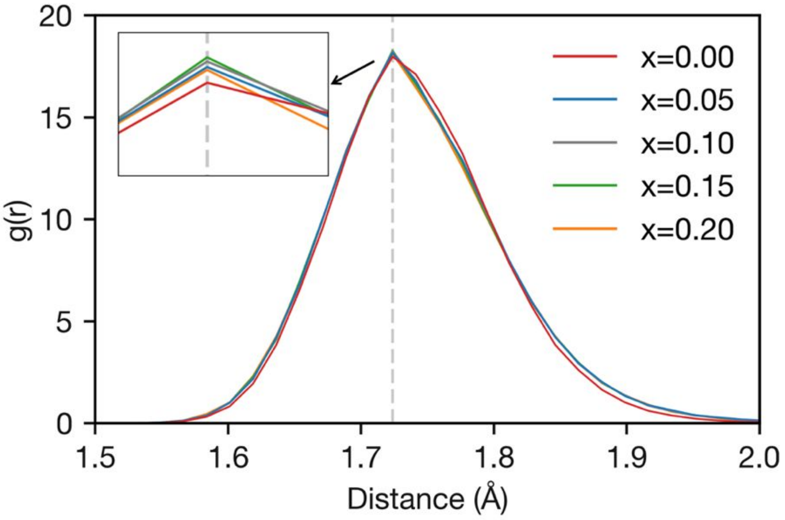

Figure S13. V-O radial distribution functions (RDFs) for the $Sr_{3-3x}La_{2x}V_2O_8$ series taken from MD simulations.

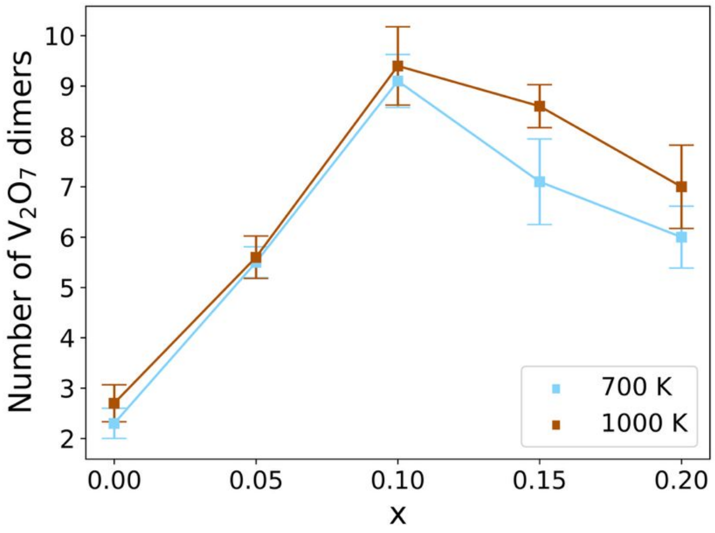

Figure S14. Average number of transient V_2O_7 dimers observed in MD simulations as a function of La content *x* at 700 and 1000 K.

References

(1) Fop, S.; Dawson, J. A.; Tawse, D. N.; Skellern, M. G.; Skakle, J. M. S.; McLaughlin, A. C. Proton and Oxide Ion Conductivity in Palmierite Oxides. *Chem. Mater.* **2022**, 34, 8190-8197.

(2) Li, M.; Pietrowski, M. J.; De Souza, R. A.; Zhang, H.; Reaney, I. M.; Cook, S. N.; Kilner, J. A.; Sinclair, D. C. A family of oxide ion conductors based on the ferroelectric perovskite $Na_{0.5}Bi_{0.5}TiO_3$. *Nat. Mater.* **2014**, 13, 31-35.
